# Systematic Review and Methodological Considerations for the Use of Single Prolonged Stress and Fear Extinction Retention in Rodents

**DOI:** 10.3389/fnbeh.2021.652636

**Published:** 2021-05-14

**Authors:** Chantelle Ferland-Beckham, Lauren E. Chaby, Nikolaos P. Daskalakis, Dayan Knox, Israel Liberzon, Miranda M. Lim, Christa McIntyre, Shane A. Perrine, Victoria B. Risbrough, Esther L. Sabban, Andreas Jeromin, Magali Haas

**Affiliations:** ^1^Cohen Veterans Bioscience, New York City, NY, United States; ^2^Department of Psychiatry, Harvard Medical School, Boston, MA, United States; ^3^McLean Hospital, Belmont, MA, United States; ^4^Department of Psychological and Brain Sciences, University of Delaware, Newark, DE, United States; ^5^Department of Psychiatry, Texas A&M University, Bryan, TX, United States; ^6^Departments of Neurology, Behavioral Neuroscience, Medicine, Oregon Institute of Occupational Health Sciences, Oregon Health & Science University, Portland, OR, United States; ^7^Sleep Disorders Clinic, VA Portland Health Care System, Portland, OR, United States; ^8^Department of Neuroscience, The University of Texas at Dallas, Richardson, TX, United States; ^9^Department of Psychiatry and Behavioral Neurosciences, Wayne State University School of Medicine, Detroit, MI, United States; ^10^Research Service, John. D. Dingell VA Medical Center, Detroit, MI, United States; ^11^Department of Psychiatry, University of California, San Diego, La Jolla, CA, United States; ^12^Center for Excellence in Stress and Mental Health, VA San Diego Healthcare System, San Diego, CA, United States; ^13^Department of Biochemistry and Molecular Biology, New York Medical College, Valhalla, NY, United States

**Keywords:** single prolonged stress, extinction retention, fear memory, animal model, prospective stress, posttraumatic stress disorder, reproducibility of results

## Abstract

Posttraumatic stress disorder (PTSD) is a mental health condition triggered by experiencing or witnessing a terrifying event that can lead to lifelong burden that increases mortality and adverse health outcomes. Yet, no new treatments have reached the market in two decades. Thus, screening potential interventions for PTSD is of high priority. Animal models often serve as a critical translational tool to bring new therapeutics from bench to bedside. However, the lack of concordance of some human clinical trial outcomes with preclinical animal efficacy findings has led to a questioning of the methods of how animal studies are conducted and translational validity established. Thus, we conducted a systematic review to determine methodological variability in studies that applied a prominent animal model of trauma-like stress, single prolonged stress (SPS). The SPS model has been utilized to evaluate a myriad of PTSD-relevant outcomes including extinction retention. Rodents exposed to SPS express an extinction retention deficit, a phenotype identified in humans with PTSD, in which fear memory is aberrantly retained after fear memory extinction. The current systematic review examines methodological variation across all phases of the SPS paradigm, as well as strategies for behavioral coding, data processing, statistical approach, and the depiction of data. Solutions for key challenges and sources of variation within these domains are discussed. In response to methodological variation in SPS studies, an expert panel was convened to generate methodological considerations to guide researchers in the application of SPS and the evaluation of extinction retention as a test for a PTSD-like phenotype. Many of these guidelines are applicable to all rodent paradigms developed to model trauma effects or learned fear processes relevant to PTSD, and not limited to SPS. Efforts toward optimizing preclinical model application are essential for enhancing the reproducibility and translational validity of preclinical findings, and should be conducted for all preclinical psychiatric research models.

## Introduction

Posttraumatic stress disorder (PTSD) is a highly prevalent and impairing condition (Kessler, 2000; Nichter et al., 2019). However, as highlighted in the Consensus Statement of the Veteran Administration PTSD Psychopharmacology Working Group (Krystal et al., 2017), there is a critical lack of advancement of pharmacological treatments to address the substantial burden of this disease. The lifetime prevalence of PTSD in the general population is ~8% (Kessler et al., 1995), making it the fifth most prevalent mental disorder in the United States (Perkonigg et al., 2000). Despite this high prevalence and costly impact, and with no Food and Drug Administration (FDA) market approvals in two decades, there seems to be no visible horizon for advancements in medications that treat symptoms or enhance outcomes in persons with a diagnosis of PTSD (Krystal et al., 2017).

Many factors have been cited as contributing to the lack of neuroscience pipelines generally, and PTSD specifically, including lack of understood mechanisms of disease, target identification and validation, predictive models, biomarkers for patient stratification and as endpoints for clinical trials, clear regulatory pathways, reliability and reproducibility of published data, and data sharing and collaboration (Jeromin et al., 2020).

Several of these challenges could be addressed with the availability of reproducible, translational, and validated animal models. However, currently, there is no well-validated animal model of PTSD, although several stress paradigms mimic the behavioral symptoms and neurological alterations seen in PTSD (Zhang et al., 2019). Reliable animal models of PTSD are difficult to establish because of the present limited understanding of the PTSD heterogeneity and of the influence of various environmental factors that trigger the disorder in humans (Aspesi and Pinna, 2019). Further, differentiating what is a model of stress vs. a model of post-traumatic pathophysiology has not been well-determined. Finally, the utility of animal models to contribute to drug development research for PTSD has been questioned given that clinically, most individuals do not succumb to PTSD following exposure to traumatic stress (Papassotiropoulos and de Quervain, 2015; Richter-Levin et al., 2019).

Even if a framework for construct validity were known, across neuroscience, the historical lack of concordance of human clinical trial outcomes with preclinical animal efficacy findings has led to a questioning of the methods of how animal studies are conducted (Macloed, 2011; van der Worp and Macleod, 2011; Steckler et al., 2015) and translational validity established.

Major global efforts have been undertaken in the past decade to address systemic issues identified in preclinical reproducibility and robustness (Steckler et al., 2015; Trust, 2015). These indicate that the most reliable animal studies are those that use randomization to eliminate systematic differences between treatment groups; induce the condition under investigation without knowledge of whether or not the animal will get the drug of interest; and assess the outcome in a blinded fashion. Studies that do not report these measures are much more likely to overstate the efficacy of interventions (Macloed, 2011). The field has also determined that fewer than one in 100 relevant publications report sample-size calculations (Sena et al., 2007). To guard against such “underpowered” studies, researchers should calculate the number of animals required to have a reasonable chance of detecting the anticipated effect given the expected variance of the data. Finally, within-study standardization is also a major cause of poor reproducibility (Voelkl et al., 2018).

As part of the Alliance for Modeling Pathological Impacts of Trauma with Unified Practices (AMP-IT-UP) program, Cohen Veterans Bioscience brought together preclinical and clinical experts in PTSD to assess existing model systems and approaches for establishing construct validity through reverse engineering, extracting from human data the constructs that could be reliably reproduced, in whole or in part, in an animal model and confirming what methods could be reliably instituted across multiple labs, including academic or industry.

Greater than 14 preclinical stress paradigms are presently in use to mimic aspects of a PTSD-like phenotypes, and these models vary extensively in their level of validation and usage, as well as the specific psychopathological features they are intended to model (reviewed in Deslauriers et al., 2018; Zhang et al., 2019). Importantly, sources of variation within the application of a preclinical model can impede the repeatability and robustness of results (Fidler and Wilcox, 2018), wasting vast resources and time. Thus, efforts to optimize preclinical PTSD models for reliability/reproducibility is essential for promoting mechanistic understanding of the disease and enhance their ability to serve as effective platforms for evaluating new and promising therapeutics (Zhang et al., 2019). Efforts to achieve methodological consensus within a preclinical PTSD model can also facilitate meta-analyses and the creation of metadata, which serve as powerful strategies in translational research (Helgheim et al., 2019).

With these overall aims, we selected the SPS model, one of the most popular paradigms in the field of preclinical PTSD modeling, combined with fear extinction retention as a test for behavioral changes relevant to PTSD to conduct an in depth methodological review to determine sources of variability and develop optimization guidelines to enhance reproducibility across laboratories. SPS is widely applied to probe multiple PTSD-relevant phenotypes (behavioral and physiological) and putative trauma mechanisms [oxytocin regulation, the neuropeptide Y (NPY) system, synaptic protein expression, and memory function] (Serova et al., 2019; Hirota et al., 2020; Liu et al., 2020; Nwokafor et al., 2020; Xiao et al., 2020). This model has a number of elements that support its adoption including: (1) SPS has defined core features that support a capacity for reproducibility (i.e., restraint for 2 h, forced swim for 20 min, and ether until loss of consciousness to promote activation of the HPA axis; Yamamoto et al., 2009; Lisieski et al., 2018), (2) the use of SPS in mice and rats, (3) its initial development to probe PTSD-specific phenotypes including glucocorticoid receptor hypersensitivity and disrupted fear extinction, (4) its stress/incubation timeline that allows manipulation at various intervention points, and (5) SPS does not rely on *post-hoc* sorting of susceptible animals (e.g., social defeat). Extinction retention is a frequently assessed outcome or end-point following SPS because individuals with PTSD and rodents exposed to SPS show fear responses to conditioned cues after a fear conditioned response has been extinguished, referred to as a deficit in extinction retention, thus making it a key target for treatment (Milad et al., 2008, 2009; Knox et al., 2012a; Perrine et al., 2016; Chen et al., 2018). These features of the model have contributed to its wide utility in the field, with a PubMed search for “PTSD” and “single prolonged stress” yielding 253 studies.

The primary aim for the systematic literature review was to focus on identifying variability that remains in the methodology of published SPS studies (Section Results of the Systematic Review of SPS Methodology) and extinction retention testing following SPS (Section Results of Systematic Review of Fear Conditioning Following SPS). Members of the AMP-IT-UP group and other SPS experts, were then invited by Cohen Veterans Bioscience to an expert panel on September 10th, 2019 to review findings and generate comprehensive methodological recommendations that can guide researchers in the application of the SPS model and further improve the model's standardization for future validation efforts (Section Results of Systematic Review of Fear Conditioning Following SPS). Given that rats are the leading animal model for SPS studies, only studies using rats were included in the systematic review and the methodological considerations focus on rats. Considerations for the application of SPS and extinction retention testing in mice are discussed in Sections Methodological Considerations for SPS in Mice and Methodological Considerations for Optimizing Fear Learning in Mice, respectively. Similar efforts to define protocols for calibrating complex outcomes under local conditions for other models could advance the field of preclinical PTSD research by facilitating the integration and replication of preclinical findings across laboratories.

## Systematic Review Methods

### Search Criteria

**Figure 1:**
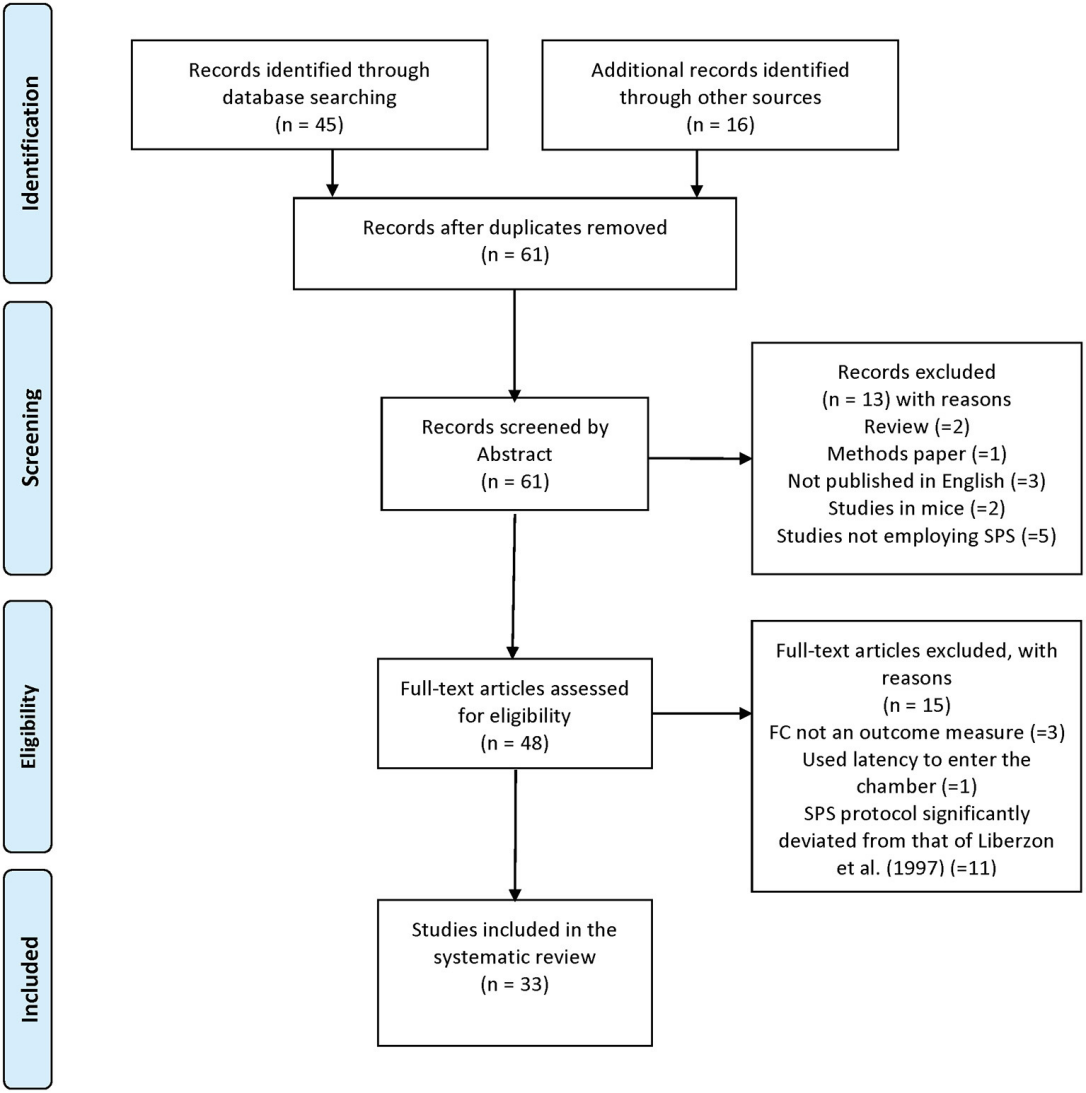
PRISMA Diagram of systematic review methods.

Our search criteria were established based on our stated aims to understand (i) how SPS is models are established and (ii) the effects of SPS on tests of extinction retention. Following the guidelines established by PRISMA (Moher et al., 2009), a comprehensive search of the PubMed database was conducted on October 1st, 2019 using the following keywords: (“single prolonged stress” OR “SPS”) AND “fear conditioning” AND (“rat” OR “rodent”); a total of 45 studies were retrieved using this search strategy (PRISMA diagram in Figure 1). Additional studies were determined by reviewing the reference lists of the included articles (*n* = 16). In total, 61 articles were retrieved and reviewed for inclusion.

### Inclusion and Exclusion Criteria

Two authors (CF-B and LC) independently screened the abstracts and titles of all 61 retrieved articles to determine whether they met the inclusion criteria. Only primary research articles examining the effect of the SPS model on extinction retention using the cued and/or contextual fear conditioning paradigms were included (two reviews and one methods paper were excluded from the dataset). Additionally, studies not published in English (3 studies), studies reporting duplicate data (1 study), studies utilizing mice as the primary animal model (2 studies), studies not utilizing the SPS model (5 studies), and studies in which extinction retention in cued or contextual fear conditioning was not examined (2 studies) or fear extinction was not the primary outcome measure (2 studies) were excluded. Additionally, studies with significant deviations in the SPS protocol first reported by Liberzon et al. (1997) in 1997 (i.e., 2 h restraint, 20 min forced swim at 24°C, 15 min recuperation and ether exposure to loss of consciousness) were also excluded as these analyses were outside the scope of this article (11 studies). Any disagreements were resolved through discussion and consensus. A final total of 33 articles met the inclusion criteria and were included in the systematic review. The full list of studies included in the systematic review is provided in Supplementary Table 1.

### Retrieval of Information From the Full-Texts

Information on the methodological details of each of the included articles was retrieved from the full texts. Two reviewers extracted the data from the 33 included studies using an excel spreadsheet. The full list of the 33 studies included is provided in Supplementary Table 1. The title, publication year, and list of authors were collected as general identifiers. The following animal and housing details were also collected: species, sex, strain, vendor, breeding site location, age/size on arrival, sample size per group, and housing conditions prior to experimental start. These details are discussed in section Methodological Considerations for Single Prolonged Stress (SPS) in parallel with recommendations from the expert panel. We also examined whether procedures were conducted during the light (active) or dark phase, and present consideration by the expert panel (Section Considerations for Timing of Behavioral Testing). For the SPS procedure, specific protocol details were extracted including: restrainer type, swim duration (min), swim water temperature (°C), single vs. group swim, duration of the recuperation period (min), compound used to induce loss of consciousness, duration of quiescent period, and details of handling procedures or disturbance parameters during the quiescent period (see section Results of the Systematic Review of SPS Methodology). For fear conditioning, extracted details included the type of fear conditioning (cued and/or contextual), descriptions of the contexts used for fear conditioning training and extinction training and extinction retention testing (i.e., the presence of visual, auditory, and olfactory cues). Additionally, we examined the interval between SPS exposure and the start of fear conditioning or any preceding behavioral tests. We also examined details of the behavioral scoring method (type: manual vs. an automated computer software; the computer scoring software manufacturer (if applicable); whether the behavior was continuously recorded vs. time sampled; the detailed information on how freezing was defined). The duration and presence of a baseline period for fear conditioning training, extinction and retention testing (s) was noted. We also considered features of the conditioned stimulus (duration, Hz, and dB), the type of conditioned stimulus (tone, light, etc.), the number of shocks, the shock duration (s), the shock intensity (mA), the intershock interval (s), and duration of the post-shock period (s). Further, detailed parameters of the extinction training and extinction retention testing procedures were recorded: the timing after fear conditioning training (h), context details, duration/presence of the baseline period (s), duration and number of conditioned stimulus presentation, and duration of the intertrial interval (s). Finally, behavioral analysis parameters were recorded and exclusion criteria.

If a study did not report a specific methodological detail it was recorded as “not reported” and were omitted from percentage calculations. Data were extracted directly from the included papers, and not from references cited within the publication. For each category and subgroup, the percentage was determined and a descriptive synthesis was performed. Results were compared for similar experimental designs. For methodological details that were infrequently reported but deemed important by the expert panel, all lead and corresponding authors were contacted to clarify methodological details for the 33 studies. Authors were contacted twice at minimum using the email listed for correspondence as well as email(s) listed on home university web-pages. Details which could not be verified were omitted from percentage calculations.

## Results of the Systematic Review of SPS Methodology

**Figure 2:**
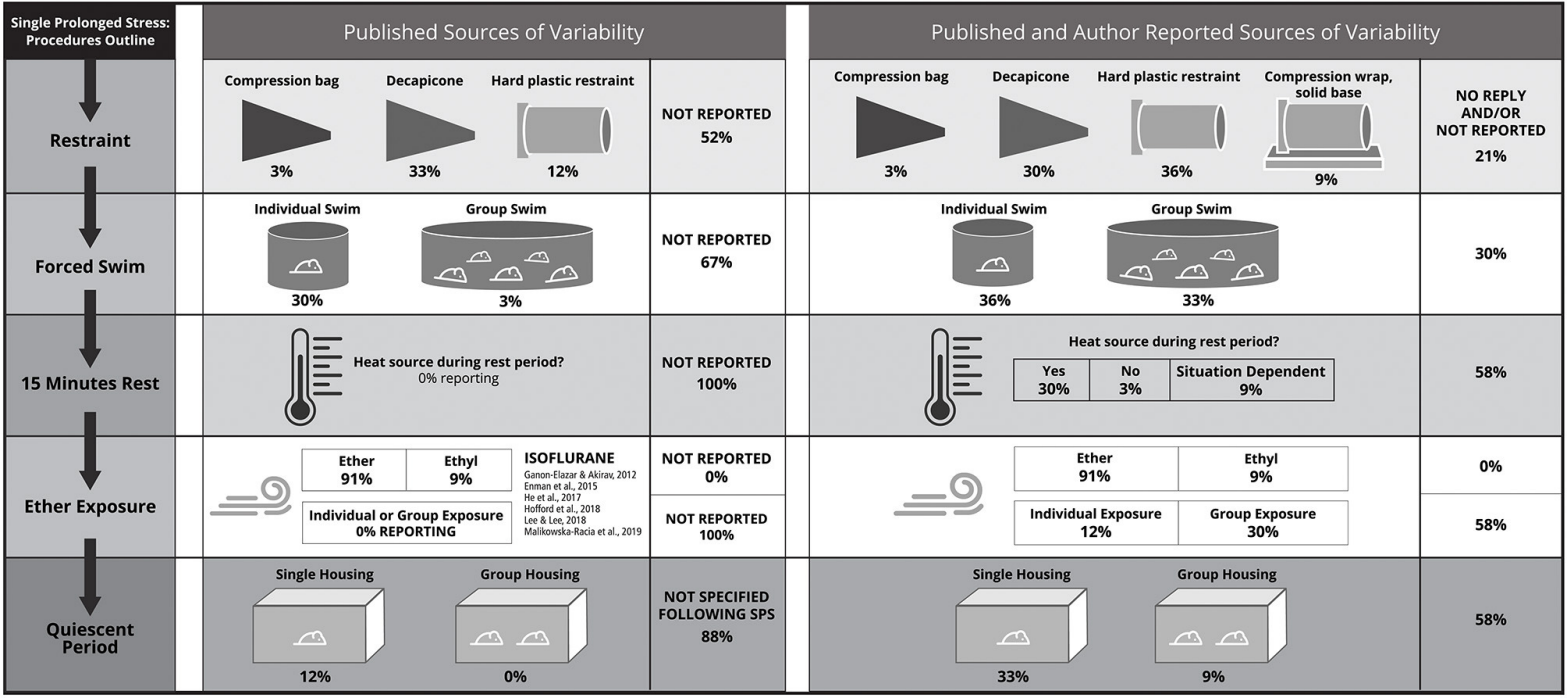
Each procedure required for single prolonged stress (SPS) is characterized by methodological variability; the chronological flow of SPS procedures detailed in the lefthand column: (i) restraint, (ii) forced swim, (iii) rest, (iv) exposure to diethyl ether vapors until the loss of consciousness, and (v) a quiescent period to consolidate effects of the SPS-stressors. Methodological variability is depicted based on published information from the 33 studies in the systematic review (central panel) and information from the systematic review expanded and refined through author contact (right-hand panel). Authors replied for only 14 studies, such that methods for only 42% of studies could be clarified. Details that were not published are denoted as “not reported” in the central panel, details that were not reported and/or could not be verified through author contact are listed in the right-hand panel as “no reply and/or not reported.” Details for the (iii) rest and (v) quiescent period are less frequently reported, but became clear sources of variability following author contact. The quiescent period was highlighted as a key source of variation by the expert panel (see section Methodological Considerations: Animal Housing). There are significant methodological differences for each SPS procedure that can contribute to lack of reproducibility, which will be subsequently described: (i) Based on published methods, restraint was most frequently applied using a decapicone, but on being directly queried, authors revealed that a number of studies used a custom-restraint type with a compression wrap and that hard-plastic restraints were used more frequently than a decapicones. This is in contrast to what was found when relying on published methods. This is critical given that animal safety considerations are specific to the restrainer type (Section Methodological Considerations: Single Prolonged Stress: Restraint Stress). (ii) The second SPS-stressor, forced swim, can be applied to individual rats or groups of rats, with author-reported group numbers varying from 3 to 8 age- and sex- matched conspecifics. This detail was omitted in the majority (67%) of published studies in the systematic review. (iii) During the 15-min rest phase, the use of heat to facilitate recovery was not reported in any of the published studies; however, this is a significant methodological consideration as heating enables rats to recover from the forced swim and before vapor exposure. Author replies indicate that a heating source was provided in at least 1 out of 3 studies or was “situation-dependent” (i.e., provided during winter but not summer); but the majority of authors failed to provide this information. Additional considerations for the rest phase include methods for drying rats following the forced swim, and whether rats are exposed to the heat source individually or in groups. (iv) There are several sources of methodological difference during vapor exposure including individual vs. group exposure and the type of anesthetic (diethyl ether, ethyl, or isoflurane). All published studies reported on type of vapor, but no study indicated whether rats were exposed to an anesthetic individually or in groups. Whether ethyl referred to diethyl ether could not be clarified by author contact. While isoflurane was not used in any of the systematic review studies, isoflurane is featured because it is occasionally substituted for ether because of logistical constraints arising from ether combustibility and personnel safety. However, isoflurane has distinct effects compared with diethyl ether and can introduce another source of variability if used in SPS (see section Methodological Considerations: Single Prolonged Stress: Ether). (v) During the quiescent period, the expert panel suggests that animals should be transferred to single-housing and “undisturbed” (see section Methodological Considerations: Animal Housing), and housing details should be reported to enhance the replicability and impact of SPS studies.

In SPS, three distinct stressors are applied in succession over the course of ~3 h, followed by a 7 day quiescent period that is required for commonly measured effects of SPS exposure to develop (Liberzon et al., 1997, 1999; Knox et al., 2012a). The SPS stressors in chronological order are restraint, forced swim, and exposure to ether vapors until loss of consciousness. Methodological variation captured by the systematic review at each stage of SPS is depicted in Figure 2. All authors were contacted for clarification around unspecified methodological details: of the 33 studies in the systematic review, 42% were clarified by author replies (14/33).

The main source of variation in the first SPS stressor, restraint, was the restrainer type. Restrainer type was not reported in 39% of studies (13/33). Similarly, the level of detail was not sufficient to identify the restrainer type in an additional 12% of studies: animal “holder” (3/33), “disposable restraint holder” (1/33). Of the studies that reported restrainer type, decapicones were used in 33% (11/33) of the studies, compression plastic bags (similar to decapicones) in 3% (1/33) of studies, rigid plastic restrainers in 9% (3/33) of studies, and a “custom-built polymethyl methacrylate individual restraining devices” (1/33). The distribution of the restrainer types differed after the authors were contacted, reflecting the importance of detailed reporting: 36% (12/33) rigid plastic restrainers, 33% decapicones (8/33) and similar plastic compression bags (3/33), 9% (3/33) compression wrap with a plastic base. The restraint type for remaining studies could not be verified, 21% (7/33). Animal safety considerations specific to the restrainer type are discussed in section Methodological Considerations: Single Prolonged Stress: Restraint Stress.

The second SPS stressor, forced swim, was applied to either individual rats or groups of rats, but this detail was omitted in 67% (22/33) of studies. In studies that reported this detail (11/33), only 1 study reported conducting the forced swim with groups of rats. However, group forced swim was a component of the initially optimized SPS procedure and the expert panel affirmed it is an important feature of SPS. After author contact, swim conditions for 10 studies still could not be verified, but group forced swim was confirmed for 33% of studies overall (11/33). Other features of the forced swim were more consistent, but were also underreported. For example, in the studies that reported the water temperature, values ranging from 20 to 24°C were reported, but 9% (3/33) of the studies did not report the water temperature. Other features of interest for the forced swim stressor that merit consideration (discussed in Section Results of Systematic Review of Fear Conditioning Following SPS) are the group size (number of animals swimming), the size of the water container, the time of day of the SPS procedures, and the use of heat during the 15 min recovery period prior to ether exposure. For example, following author contact, it was determined that rats were provided with a heat source during the 15-min recovery period in 93% of studies that could be clarified.

For the third SPS stressor, the majority of studies used (diethyl) ether as the anesthetic (91%; 30/33). The remaining studies reported “ethyl” (9%; 3/33; Lin et al., 2016a, 2019a,b), which could refer to diethyl ether but could not be clarified. Other features of interest for the ether exposure stressor that merit consideration [discussed in section Methodological Considerations for Single Prolonged Stress (SPS)] are whether the rats are exposed to an anesthetic individually or in groups, the size of the anesthetic chamber, and the method for verifying loss of consciousness (i.e., toe pinch, righting response). In the 14 studies that were clarified by authors, it was determined that 71% of studies (10/14) exposed rats to ether in groups. Of note, the phrase “loss of consciousness” is used throughout the publications in the systematic review, however consciousness (or its loss) cannot be established in a rodent. Some recent descriptions of ether exposure in the context of SPS have indicated the ether stressor was terminated when “general anesthesia” was induced, reflecting the inability to assay consciousness (Knox et al., 2016; Moulton et al., 2018).

Following administration of the SPS stressors, a 7 day delay is necessary for key behavioral and neurobiological manifestations of SPS to develop (Liberzon et al., 1997, 1999; Knox et al., 2012b). For example, the effects of SPS on glucocorticoid receptor expression and glucocorticoid negative feedback emerge after a 7 day quiescent period (Liberzon et al., 1997, 1999; Knox et al., 2012b). Additionally, many cellular effects of SPS are transient or dependent on time and context (Souza et al., 2017; Serova et al., 2019). Housing during the quiescent period was reported in only 12% of studies, and all reporting studies single-housed the animals during the quiescent period. Following author contact, it was determined that 79% (11/14) of studies single-housed the rats following SPS. The duration of post-SPS recovery was variable, but the majority (76%; 25/33) of studies reported a 7 day period following SPS in which rats were not manipulated, the post-SPS recovery duration for which SPS was initially optimized (Liberzon et al., 1997, 1999). One study omitted the quiescent period and implemented fear conditioning procedures the day following SPS (Mirshekar et al., 2013), while another compared a 1 and 7 days quiescent period (Kohda et al., 2007). Four studies implemented drug injections during the quiescent period (Miao et al., 2014; George et al., 2015; Lin et al., 2016b; Liu et al., 2016). In two studies, the delay prior to outcome testing was unclear (Imanaka et al., 2006; Han et al., 2017). An additional two studies used longer quiescent periods: 10 days (RaiseAbdullahi et al., 2019) and 14 days (Takahashi et al., 2006). The delay between SPS and outcome testing was also variable, but most studies (64%; 21/33) tested fear conditioning following a 7 day quiescent period; the timeline for which SPS was initially optimized (Liberzon et al., 1997, 1999; Knox et al., 2012b). Some publications included more than one timeline for testing fear conditioning after SPS, such that they are represented more than once in the presented timeline summaries. Of the remaining experimental timelines, most used longer delays before outcome testing: 10 days (1/33), 14 days (5/33), 16 days (1/33), and 28 days (3/33). Finally, two experiments started fear conditioning the day after SPS (Kohda et al., 2007; Mirshekar et al., 2013).

## Results of Systematic Review of Fear Conditioning Following SPS

SPS has been in use for over two decades to evaluate the effects of trauma across a number of outcome domains, including behavior/cognition outcomes (extinction retention, fear recall, startle responsivity, anxiety, anhedonia, cognitive flexibility), neuroendocrine function (corticosterone and catecholamine plasma levels, and correspondent receptor protein levels in the brain), synaptic plasticity (spine density/frequency, synaptic protein levels), gene expression, the inflammasome, sleep, and ethanol and drug consumption (reviewed in Souza et al., 2017; Lisieski et al., 2018). A limitation of the SPS model is that “seemingly subtle deviations in the procedure may have significant consequences on (resulting) behavior and physiology” (Souza et al., 2017). This challenge is compounded by omitted or variable methodological details. Given the prevalence of extinction retention testing following SPS, in this section, we detail the variability in extinction retention testing parameters following SPS. The advantages of extinction retention as a preclinical outcome measure include its non-invasiveness, non-lethality, and flexibility in being combined with other outcomes of interest. Limitations include unclear repeatability in the same animal, necessary training, non-learner attrition, costly equipment for implementation, and dependence on freezing as a proxy for fear given that freezing may be less suitable for females and younger animals (Shansky, 2015; Bangasser and Wicks, 2017; Graham et al., 2018). Additionally, it is unclear the degree to which freezing as a proxy for fear translates to the human condition, i.e., few studies have prospectively examined whether defensive reactions such as freezing play a role in the development of psychopathologies such as anxiety disorders given that most studies in humans rely on retrospective self-reports of freezing/immobility related to experiencing trauma or flashbacks (reviewed in Roelofs, 2017).

***Pop-Out 1: Implications of the systematic review for a meta-analysis of SPS***.

*This systematic review revealed that key methodological details are frequently omitted from study reports for each SPS stressor and for the housing conditions following SPS. This has multiple implications for interpreting results and can also impede the interpretation of findings when conducting a meta-analysis of published SPS literature. While meta-analyses are a powerful tool for cumulating and summarizing knowledge in a scientific field, the power of a meta-analysis, as outlined by the Quality of Reporting of Meta-analyses (QUOROM), depends on bringing together results across multiple studies, that may be individually small or underpowered, to detect a statistically significant outcome (Russo*, *2007**; Forero et al.*, *2019**). Data extraction is a key step in this process and may require directly contacting authors when information is missing, but author response is typically lower than expected. This provides a considerable challenge in that excluding papers due to missing data may distort outcomes from a meta-analysis (Russo*, *2007**; Schmucker et al.*, *2017**). The inclusion of the methodological details discussed here is important to enhance research robustness and reproducibility and enhance the advantages of preclinical research through improving internal control*.

**Figure 3:**
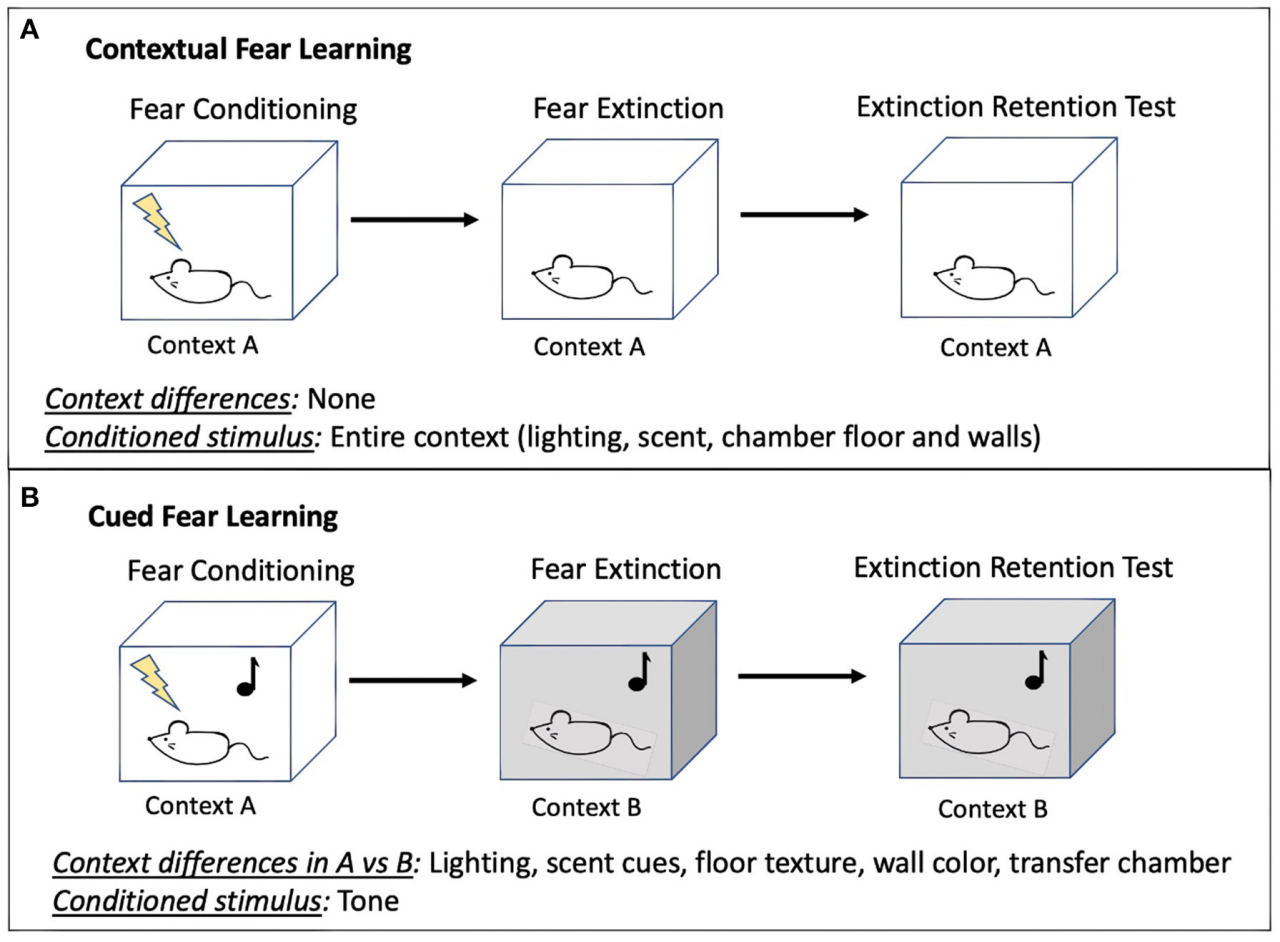
Diagram of contextual **(A)** and cued **(B)** fear learning methodologies. Variability in behavioral results during each phase of fear learning can derive from features of the conditioned and unconditioned stimuli, testing environment, housing conditions, time of day at testing, as well as animal features such as age, sex, and baseline fear behaviors (discussed in section Methodological Considerations for Fear Conditioning Following SPS). Note, although a tone is often used as the conditioned stimulus to pair with shock for cued fear conditioning, other cues may be used if they are distinct from other contextual features, discrete, and repeatable.

To measure extinction retention, researchers can use contextual (42% of studies; 14/33) or cued fear (45% of studies; 15/33). Some SPS studies (12%; 4/33) used a combination of these methods. In contextual conditioning (Figure 3A), an aversive stimulus is paired with a context (day 1: fear conditioning). The context is often characterized by a distinct odor, wall color, floor texture, or lighting condition. Animals are then re-exposed to the context without the shock to extinguish the fear response (day 2: fear extinction). Finally, animals are exposed for a third time to the context to test their retention of the extinction memory in comparison with the initial fear memory (day 3: extinction retention). In cued conditioning (Figure 3B), animals are first trained to associate a neutral (conditioned) stimulus with an aversive (unconditioned) stimulus through repeated pairings of the unconditioned and conditioned stimulus, referred to as fear conditioning (day 1: fear conditioning). Then, in a novel context, animals are repeatedly presented with the conditioned stimulus until fear responses to the conditioned stimulus are extinguished (day 2: extinction training) (Maren, 2014). The fear conditioning context is distinguished from the extinction context using a variety of contextual cues which can be visual, olfactory, and tactile (discussed in section Considerations for Optimizing Conditioned Fear Behavior). Finally, animals are returned to the second context and re-exposed to the conditioned stimulus to test extinction memory in comparison with initial fear memory (day 3: extinction retention). In both contextual and cued paradigms, an extinction retention deficit is defined by heightened fear expression during re-exposure to the extinction context, despite fear behavior having decreased over the course of extinction training, suggesting that fear memory dominates the competing extinction memory (Bouton and Bolles, 1980; Milad et al., 2007; Lonsdorf et al., 2019). Variability in results can derive from experimental conditions including features of the conditioned and unconditioned stimuli, testing environment, housing conditions, time of day at testing, as well as animal features such as age, sex, and baseline fear behaviors. Given these diverse influences, attempts at replication of previously published conditions may not yield interpretable extinction retention results because local conditions must be adjusted to optimize extinction retention performance. Methodological considerations for optimizing local conditions for testing extinction retention are included in section Methodological Considerations for Fear Conditioning Following SPS.

The systematic review demonstrated that the unconditioned stimulus used following SPS was consistently a foot shock, with substantial variability in features of the shock including comparisons of different shock features within studies in the systematic review (variation detailed in Table 1). For example, the number of shocks varied significantly across the 35 distinct experimental designs in the 33 studies: 1 shock (30% of studies), 2 shocks (9% of studies), 3 shocks (6% of studies), 5 shocks (33% of studies), 7 shocks (15% of studies), 8 shocks (3% of studies), and 10 shocks (6% of studies), with 1 study not reporting the number of shocks. Nearly half (49%; 17) of the 35 experimental designs used 1 mA as the shock intensity, but the intensity ranged from 0.3 mA to 1.5 mA. The shock duration was 1 s in 46% (16/35) of designs, but ranged from 2 s to 30 s. The conditioned stimuli targeted several sensory modalities, including visual (a light; 15% of studies; 5/33) and auditory (a tone; 42% of studies; 14/33), or the combination of all contextual cues in 39% of studies (13/33); not specified in 1 study. For studies using an auditory cue, 64% used a 10 s tone duration (9/14), while 36% used a 30 s tone duration (5/14). The frequency 2 kHz was used in 57% of studies using a tone (8/14), but other frequencies included 1 kHz (2 studies), 3 kHz (1 study), 4 kHz (2 studies), and 9 kHz (1 study) were also used. The tone decibel was 80 dB in 79% of studies using a tone (11/14), with the remaining studies using 70 dB (1 study) or 75 dB (2 studies).

**Table 1:**
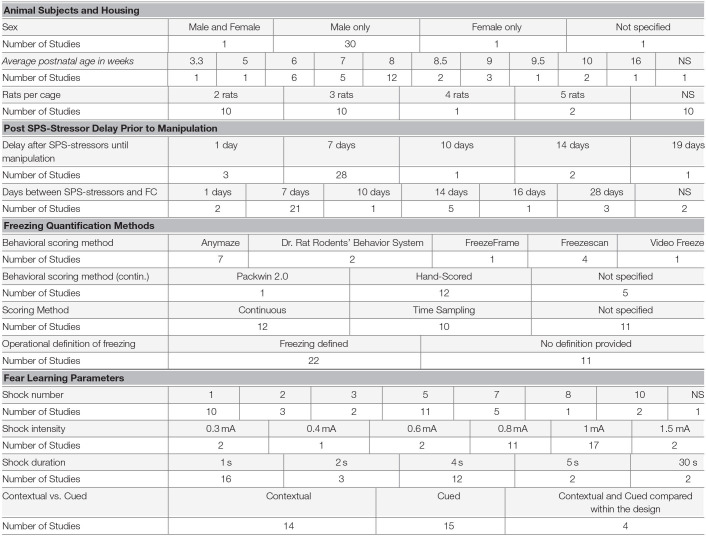
Variability in methodological details reported in systematic review publications.

*NS, Not specified*.

*Studies = Number of studies in the systematic review that reported this feature*.

*Please note the above table represents procedural details as they are represented in the publications included in the systematic review*.

For fear extinction training in contextual paradigms, the duration of re-exposure to the context for extinction ranged from 5 to 20 min. For studies using cued fear extinction, over half (53%) used 30 trial blocks (9/17); each block was generally comprised of a 10 s cue presentation and 60 s intertrial interval (ITI), for a total trial time of 35 min. The remaining cued fear paradigms used 15 trial blocks, with the exception of 1 study, Noble et al. (2017), which extinguished fear learning with 4 tone presentations in the absence of shock each day for 11 days with random ITIs ranging between 120 and 240 s. Whether the ITI is a fixed or variable duration can affect the robustness of the tone-shock association (Badia et al., 1975). Both fixed and variable ITIs have been used following SPS: in the cued studies evaluated here, 79% used fixed 60 s ITIs and 16% used variable ITIs (15/19 and 3/19, respectively). The remaining study used a single cue presentation followed by 120 s of behavioral monitoring (Han et al., 2017). The duration of extinction training is generally longer in cued paradigms compared with contextual paradigms, which likely reflects the higher number of shocks used during cued fear conditioning (average of 5.8) vs. contextual fear conditioning (average of 2.1 shocks). Higher numbers of shocks generate stronger fear associations, which require more thorough extinction training (see section Considerations for Optimizing Conditioned Fear Behavior). The duration of extinction retention testing should be sufficient to allow for a comprehensive test of extinction recall following acclimation from transport/handling stress and the novel context. In the studies evaluated, extinction retention testing ranged from 3 to 12 min, and was generally shorter in contextual paradigms.

Variation in how extinction retention is operationally defined, measured, and statistically analyzed detracts from the robustness and translatability of this measured (Lonsdorf et al., 2019). For example, studies using cued fear extinction retention testing with 10 cue presentations reported a variety of statistical methods to evaluate freezing during extinction retention. Within the studies evaluated, approaches have included the evaluation of freezing in individual trials, blocks of 2–4 trials, and subsets of trials separated across early and late phases of extinction retention. For example, a repeated measures analysis of variance (RMANOVA) of baseline and all 10 individual trials was used in Chen et al. (2018), while a RMANOVA was applied on 10 individual trials, without including baseline freezing, in Harada et al. (2008). Patterns of trial blocking have also been used for cued extinction retention testing across 10 trials, including blocks of 2 and 4 trials (Keller et al., 2015a). Comparisons within and across an early and late phase of testing have also been used, reflecting the secondary extinction process that occurs during extinction retention as animals are repeatedly re-exposed to the conditioned cue across the extinction retention trials. For example, an RMANOVA on trials separated into an early phase (first 5 trials) and late phase (last 5 trials) was used in Chen et al. (2018) and Chaby et al. (2019). Similarly, trials were averaged within an early and late phase and measured with separate ANOVAs (George et al., 2015). Knox et al. (2012a,b) also averaged trials within an early and late phase and compared across phases using a two factors design.

Baseline freezing, prior to the first extinction retention cue presentation, has been analyzed separately and can be increased by SPS (George et al., 2015) or not affected (Knox et al., 2012b; Keller et al., 2015a). To account for potential individual differences in baseline freezing, researchers have calculated extinction indexes by subtracting baseline freezing from the average percent freezing across 10 cued extinction retention trials (Knox et al., 2012b). In humans, extinction retention indexes have been used to account for individual differences in the strength of the fear association acquired during cued fear conditioning (Milad et al., 2007, 2009; Rabinak et al., 2014; McLaughlin et al., 2015) and the strength of cued extinction learning (Rabinak et al., 2014).

In contrast with the cued fear conditioning studies evaluated, some studies using contextual fear conditioning used repeated days of extinction training to assess retention across multiple exposures. In these studies, freezing was averaged within each day and analyzed with a RMANOVA or two-way ANOVA across days (Yamamoto et al., 2008; Matsumoto et al., 2013; Kataoka et al., 2018). Representative values for a trial day are generated using variable methodologies: the percentage of time generated using sampling over time with categorically handscoring of freezing (Kohda et al., 2007), percentage of time yielded by a continuous automated software (Harada et al., 2008), or total seconds spent freezing (Imanaka et al., 2006; Iwamoto et al., 2007). Variability in data processing, trial blocking, and statistical analysis complicate meta-analysis efforts, such that it is challenging to effectively compare results of studies and generate effects size estimates despite similar methodologies.

Another source of variability is the method for the detection of behavior during the trials (detailed in Table 1). Freezing behavior is quantified as a proxy for fear using manual scoring (36% of studies; 12/33), automated software (48% of studies; 16/33), or not specified in 5 studies (15%). Operational definitions of freezing were variable and provided in only 67% of studies (22/33), but were often explained as complete immobility except for movement necessary for respiration. Variability in freezing measurements, from the same experimental conditions, can derive from differential detection methods. For example, continuous vs. time sampling measurements, variation between scoring software, the operational definition of freezing, and the use of exclusion criteria (considerations detailed in section Recommendations for Freezing Detection and Data Analysis). Overall, 33% of studies did not state whether the freezing analysis was continuous or used a time sampling approach (11/33). Of those that did specify, 55% used continuous analysis and 45% used time sampling (12/33 and 10/33, respectively). Several software packages were used across the 33 studies evaluated: Anymaze (25%), Freezescan (14%), Dr. Rat Rodent's Behavior System (7%), Packwin 2.0 (4%), Freezeframe (4%), and Video Freeze (4%). Software packages vary in the level of validation for the detection of freezing and the number and role of automated vs. user-determined thresholds to define freezing. These features result in differential relationships between software vs. manually coded freezing behavior (Haines and Chuang, 1993; Marchand et al., 2003; Anagnostaras et al., 2010). Despite the high variability that can derive from software thresholds (Luyten et al., 2014), threshold settings are only occasionally reported (for example in fear conditioning following SPS). There are other software features that can also affect the concordance between freezing measure detected manually or using software, including whether background subtraction is used (Marchand et al., 2003) and the quality of the video recording (frames per second, lighting, background contrast, camera resolution, etc.; Pham et al., 2009), which were also rarely reported. These variables can be disseminated through published protocols, supplementary methods, or recorded in internal laboratory protocol documents to ensure consistency between experiments within a lab. Variability in software settings can determine whether or not group differences are detected (Luyten et al., 2014), and therefore it is difficult to assess the degree to which freezing quantification methods contribute to variability across SPS studies with the current level of detail in reporting. Meuth et al. (2013) tested the differences in freezing measurements across laboratories by providing laboratories with the same fear extinction videos to be evaluated under local conditions. They found that some discrepancies between laboratories in percent freezing detection reached 40% between observers, and discordance was high for both manual and automated freezing detection methods.

Concerns over inter-lab variability in fear conditioning methodologies have spurred recent calls for standardized methodological recommendations for fear conditioning procedures (Wotjak, 2019). Efforts to standardize methodological reporting and how extinction retention data are analyzed and disseminated could advance efforts to comply with NIH mandates related to robustness and reproducibility as well as enhance the potential for translation of preclinical results (Baxter and Burwell, 2017). To facilitate replicability and the potential for meta-analytical efforts to advance the field, published reports should detail (i) manual vs. automated freezing detection methods, (ii) the operational definition of freezing, (iii) continuous vs. time sampling methods, (iv) software thresholds, if applicable, (v) video recording quality, as well as the features of the experimental conditions detailed in section Methodological Considerations for Fear Conditioning Following SPS. Similar efforts to define sources of variability could be conducted for other preclinical models of psychiatric illnesses to advance the robustness and rigor of preclinical research.

## Methodological Considerations for Single Prolonged Stress (SPS)

### Methodological Considerations: Animal Subjects

Even though preclinical models of psychiatric disorders cannot recapitulate the entire spectrum of symptoms and behavioral characteristics present in these disorders, a key advantage of the preclinical approach is the degree of internal control that can be leveraged to establish causality and characterize mechanisms that shape pathological outcomes. To maximize this advantage, sources of variation within preclinical research need to be minimized. A key source of variation in preclinical research is the origin of the animals used; variation in stress neurobiology, physiology, and behavior has been documented extensively across rat strains (Miller et al., 1968; Dhabhar et al., 1997; Gómez et al., 1998; Faraday, 2002; Cohen et al., 2006). Additionally, there is variation between commercial vendors within rat strains including Sprague Dawley (Pollock and Rekito, 1998; Fitzpatrick et al., 2013), between breeding sites (colonies) within vendors (Bueno et al., 2003; Fitzpatrick et al., 2013), and as a result of shipment and the age at shipment (Fontoura-Andrade et al., 2017). Sprague Dawley, an outbred strain of rats, are most frequently represented in the literature and account for 76% of studies on SPS. Further, the predominance of Sprague Dawley rats has been consistent over the last two decades and across fields including addiction research (Liberzon et al., 1997; Wang et al., 2008; Eagle et al., 2015; Yu et al., 2016). Consistency in rat strain is beneficial for cross-study comparisons, as strains differ in responsivity of the hypothalamic-pituitary-adrenal (HPA) axis following SPS as well as other psychophysiological stressors including a predator-based preclinical model for a PTSD-like phenotype (Dhabhar et al., 1997; Cohen et al., 2006; Malkesman et al., 2006). Compared with rat strain, there is greater variability in animal sourcing at the level of the vendor and site, as well as reduced reporting. Greater than 1 in 10 studies evaluated did not report their animal vendor. In studies that reported the vendor (28/33 studies), 52% sourced rats from Charles River, but the remaining 48% of studies were distributed across 7 additional vendors. Vendor site was more variable and less frequently reported, and was not specified in 39% of studies (13/33 studies). Of the SPS studies in the systematic review that report vendor site (20/33 studies), the Charles River site in Yokohama, Japan accounted for 21% of studies (7/33 studies). An additional 9 sites made up the remaining 40% (13/33) of reporting studies, such that vendor site varied greatly across studies within and across countries represented (China, Japan, Iran, the United States, and Taiwan).

***Pop-Out 2: General considerations for optimizing repeatability of preclinical results***.

*As with all preclinical experiments, there are general methodological considerations that can be applied to SPS studies to enhance to enhance robustness and repeatability (Kilkenny et al.*, *2009**). For example, animals should be assigned to groups randomly or balanced for prognostic factors, and housed randomly or with block randomization (Collins and Tabak*, *2014**). For studies involving the application of stress, stress-exposed and non-stressed animals should not be housed within the same cages and care should be taken to minimize scent-transfer because stress manipulations can elevate aggressive behavior and alter pheromone signals (Kikusui et al.*, *2001**). Specific recommendations for SPS housing are discussed in section Methodological Considerations: Animal Housing*.

*Uncontrolled variability can shape experimental outcomes, thus experimenters should take efforts to minimize the variability of animals between and within cohorts by maintaining a shared laboratory document that is easily accessible to all researchers in a laboratory detailing animal features and conditions (vendor, site, duration of habituation, age at testing, husbandry details). Experimental waves and cohorts should be balanced by treatment and testing order should be randomized or block randomized (Kilkenny et al.*, *2009**; Festing*, *2014**). To minimize experimenter-related factors, experimenters should be blind to treatment while administering all procedures and the experimenter should ideally not be present in the room during behavioral/outcome testing (i.e., use video monitoring). Such efforts ensure consistency within and across experiments and researchers, and can minimize the impact of variability despite turnover of laboratory personnel*.

*Although evaluation of whether animal strain and vendor site affects outcomes is beyond the scope of this review, there are key differences between strains and sites (Bueno et al.*, *2003**; Fitzpatrick et al.*, *2013**), as well as age at shipping (Fontoura-Andrade et al.*, *2017**), and reproducibility can be enhanced by reporting animal features and providence. Thus, detailed methodological reporting including animal vendor/breeding site will minimize the impact of animal source variation across preclinical research, thereby facilitating replication and expansion of the field over time. Additional information around optimal details to report for preclinical research are provided by the ARRIVE Guidelines (Kilkenny et al.*, *2009**; Percie du Sert et al.*, *2020**)*.

### Methodological Considerations: Sample Size

Low statistical power (because of low sample size of studies, small effects or both) negatively affects the likelihood that a nominally statistically significant finding actually reflects a true effect (Button et al., 2013). Under-powered studies have increased risks of selection bias resulting from baseline characteristics of animals represented across groups, detection bias, and adverse effects of attrition (Hegedus and Moody, 2010; Hooijmans et al., 2014). Thus, it is recommended that SPS studies determine group sizes with a power analysis for the specific outcomes of interest, with a minimum of 12–15 animals per group based on known variability in responses to stress (Saur et al., 2016). Further, larger group sizes enable the investigation of individual variability in susceptibility to effects of SPS and stratification for high and low responder groups (e.g., Ying et al., 2016; Serova et al., 2019). For example, Serova et al. (2019) included ~50 rats per group combined from 3 separate experiments to allow for group stratification by anxiety level and the characterization of a maximal anxiety group. Early evidence from other complex behaviors supports this sample size to compare high and low responder groups (Belin et al., 2011) or stratify individual trajectories (Chen et al., 2012). Over the course of many experiments in consistent conditions laboratories can generate response distribution curves, which can enable the categorization of individual responses by comparison to established response curves rather than only other individuals within that cohort, as has been done for a predation-based model of traumatic stress (Cohen and Zohar, 2004) and other complex behaviors (Fitzpatrick et al., 2013). Researchers are encouraged to take advantage of available tools for developing and reporting of statistical analysis plans including CAMARADES and ARRIVE.

### Methodological Considerations: Effects of Sex

Bias in the representation of biological sex is a challenge across preclinical research (Zucker and Beery, 2010; Zakiniaeiz et al., 2016). This bias is particularly concerning in neuropsychiatric research given the disparities between males and females in the incidences of various mental illnesses. For example, women are two to three times more likely to develop PTSD compared with men (reviewed in Olff, 2017). Currently, 94% of SPS experiments represented in the literature that report the sex of animals tested use only male rats (3% not specified). This bias is present across other preclinical models of preclinical models of severe stress and PTSD-like phenotypes. For example, in an identical number of studies utilizing predator cues to model features of PTSD, of those that reported the biological sex of the animals, 97% used only male rats (3% not specified). Preliminary studies have documented sex-specificity in behavioral and neurological outcomes following SPS, e.g., females do not consistently exhibit the effects of SPS on extinction retention (Keller et al., 2015b; Ornelas and Keele, 2018; Pooley et al., 2018a,b; Nahvi et al., 2019; Nwokafor et al., 2020). However, females are equally sensitive to SPS effects on depressive-like behavior, increased anxiety, and elevated hippocampal glucocorticoid receptor expression (Keller et al., 2015a; Nahvi et al., 2019). Additional efforts are underway to expand the application of SPS to female rats, in response to sex-specific biological findings and initiatives by primary funding agencies (reviewed in Clayton, 2018). These efforts are informed by sex-specific findings in other preclinical models of PTSD, as well as sex-specificity in learned fear behavior and many other prevalent assays used with preclinical trauma models (reviewed in Shansky, 2015).

### Methodological Considerations: Effects of Age

The SPS model was initially conceived over two decades ago using male Sprague Dawley rats weighing between 180 and 350 g, or ~6 to 11 weeks of age (reviewed in Lisieski et al., 2018). Currently, age is highly variable in SPS research; the most frequent age of SPS exposure, 8 weeks, was found in 36% of studies (91% of the studies reported using rats between 6 and 11 weeks of age). Age-specific outcomes to trauma have been documented in clinical populations and are of interest to preclinical modeling (Green et al., 1991; Chen et al., 2018; Cross et al., 2018), yet early efforts to apply the SPS model to younger developmental stages have found that juvenile and adolescent rats have an apparent resilience to the effects of SPS on extinction retention (Chen et al., 2018). This difference may reflect age-specific behaviors and fear responses; younger rats have differential levels of locomotor activity, stress responsivity, and are susceptible to different predator species compared with adult rats, driven by body size as well as territory expansion in adolescence (Davis, 1953; Wiedenmayer and Barr, 2001; Lupien et al., 2009; Feng and Himsworth, 2014). Developmental stress history can modulate the effects of adult trauma models, for example, juvenile stress exposure increased susceptibility to an animal model of PTSD that is based on acute swim stress and predator odor (Avital and Richter-Levin, 2005; Horovitz et al., 2012). Age-specific fear behaviors may be difficult to measure in younger animals using outcomes optimized for adults (Bronstein and Hirsch, 1976; Wiedenmayer and Barr, 2001). Additional age-specific logistical challenges exist for SPS and fear conditioning outcomes. For example, rats larger than 350 g may have limited mobility in fear conditioning chambers, which artificially elevates freezing, or they may have excess fat stores that facilitate floating during the forced swim. Overall, the application of SPS to developmental stages prior to adulthood and aged populations may require additional optimization.

### Methodological Considerations: Animal Housing

#### Prior to SPS

Animals should be socially housed prior to SPS in groups of 2–4, depending upon the size of the animals and home cage, according to the Guide for the Care and Use of Laboratory Animals, 8th edition [National Research Council (US) Committee for the Update of the Guide for the Care Use of Laboratory Animals, 2011]. Continuous single housing should be avoided, as prolonged social isolation in humans and non-human animal species can lastingly affect stress response systems that overlap with systems implicated in PTSD and the response to trauma, including inflammation, glutamatergic activity, and HPA axis function (reviewed in Cacioppo et al., 2011). In studies that reported the animals' housing conditions, the majority conformed to group housing; ~1 in 3 studies housed the rats in groups of 2 and an additional 1 in 3 studies housed the rats in groups of 3. However, 60% of the studies do not report housing conditions prior to the initiation of SPS.

#### Post SPS

Following SPS, 12% of studies socially isolated the rats, while the remaining 88% of studies did not specify the post-SPS housing conditions. An absence of social support can exacerbate the adverse effects of stress in humans and in rodent models (humans; Ozbay et al., 2007; rats; Weiss et al., 2004; Zlatković et al., 2014), such that social isolation following trauma augments PTSD incidence. As reported above, it is also a key feature of the SPS paradigm and it is recommended that rats be socially isolated for 7 days immediately following the SPS stressor day to consolidate the effects of SPS. A 7 day sensitization is necessary for cornerstone SPS effects to develop. For example, the effects of SPS on HPA regulated negative feedback, glucocorticoid receptor mRNA expression, and extinction retention are only evident after a 7 day quiescent period (Liberzon et al., 1997, 1999; Knox et al., 2012b). Thus, it is recommended that isolation continue for at least a week following the SPS stressor day and through outcome testing, and that outcome testing commence at the earliest on the 8th day following the SPS stressor day.

To ensure SPS effects, during the 7 day quiescent period, rats should be isolated and “undisturbed.” Undisturbed housing conditions are defined by the following features: (1) an absence of handling; (2) minimal research and husbandry personnel entries into the housing room; (3) an absence of cleaning or replacing caging; (4) refraining from feeding animals, enabled by providing sufficient food for the quiescent period on the day of SPS. To account for cage cleaning across SPS and control groups, all animals should be transferred to clean cages on the SPS stressor day. To minimize personnel entries into the housing room, experimenters can house SPS animals in a separate area or room from control animals. Housing SPS and control animals separately after SPS can also mitigate the effects of ether vapor or stress-induced scent cues in the housing room, as ether will continue to evaporate from the fur of the SPS animals for several hours after SPS. However, if animals are housed separately, thorough measures should be taken to standardize conditions across the two rooms. Housing SPS and control animals in the same room accounts for potentially confounding disturbances while separate housing rooms may be subject to confounding intrusion errors due to environmental features that are not standardizable or perceptible by experimenters (differential vibrations from climate control systems, neighboring mouse colony or testing rooms, etc.; Hurlbert, 1984; Hooijmans et al., 2014). Additionally, given that each experimental group must be represented in each wave of outcome testing, the logistics of sourcing animals from two rooms should be considered throughout the experimental design.

#### Control Animals

To account for disturbances necessary to implement the SPS procedures, control animals should be removed from the housing colony and placed in a novel room for the duration of the SPS procedure. Similarly, to account for housing effects, all rats should be provided clean caging on the SPS stressor day and “undisturbed” according to the above definition. Given that isolation is necessary for key SPS effects, it is recommended that control animals also be isolated during the quiescent period to distinguish the effects of SPS from those of social isolation (Raz and Berger, 2010). The importance of accounting for the effects of isolation across treatment groups is emphasized by evidence that isolation is capable of modulating HPA axis activity and immunoreactive cells, with potentially more extreme effects in younger animals as well as variation across strains and stress history (Malkesman et al., 2006). Any deviation from the standard SPS procedure should be described in the publication.

### Methodological Considerations: Animal Handling

The frequency of handling is a source of variation in preclinical research that modulates baseline anxiety-like behavior, stress reactivity, and the effects of anxiolytic drugs (Hurlbert, 1984; Daskalakis et al., 2011; Biggio et al., 2014; Hodges and McCormick, 2015). The behavioral and neuroendocrine effects of handling demonstrate that handling methods merit description in preclinical publications. For preclinical studies using animal models of stress or trauma exposure, a minimum of 7–10 days of habituation in the housing facility is recommended before the start of any procedures, handling each animal for ~2–5 min per handling session at least once per week starting ~3–4 days after the animals arrive at the laboratory facilities. Additionally, animals can respond differently to handlers based on the handler's gender, odor, or technique (reviewed in Burn, 2008), such that handlers should be consistent between cohorts and thoroughly trained to standardize techniques. These recommendations also reflect findings that handling can lessen the hyperarousal effects of transport or manipulation in laboratory rodents (Drozdowicz et al., 1990; Swallow et al., 2005). Thus, with frequent handling (i) stress-manipulations are less likely to interact with stress-responses initiated prior to an experimental procedure, and (ii) assessments that rely upon fear extinction will not have to overcome stress-responses initiated by handling.

### Methodological Considerations: Single Prolonged Stress

The SPS model exposes rats to three stressors in succession. First, rats are restrained for 2 h, which is followed by 20 min of forced swim. After swimming, rats are dried and allowed to recuperate for 15 min. Finally, rats are exposed to ether vapors until loss of consciousness. Considerations for each phase are discussed below and summarized in Table 2. The outcomes of SPS have been optimized for SPS exposure at the early stage of the inactive phase of the circadian cycle (i.e., the light phase for nocturnal rodent species). Exposure to stressors in the dark cycle, including forced swim, have reduced effects on behavior, serum corticosterone, and adrenal ascorbic acid concentrations compared with exposure during the light cycle (Kelliher et al., 2000).

**Table 2:** Methodological considerations for SPS in rats to guide experimental design and methodological reporting.

**Experimental Feature**	**Methodological Recommendations**	**Additional Considerations**
Experimental Conditions	Standardize time of day for the application of all experimental procedures, including SPS	Testing during either the light (inactive) or dark (inactive) phase; discussed in Considerations for Timing of Behavioral Testing
		Treatment of control animals not exposed to SPS; discussed in Control Animals
		Testing personnel; discussed in pop-out “Research personnel as part of an experimental context”
Housing Conditions	Group housing prior to SPS; age- and sex-matched; see Prior to SPS	Housing should be in accordance with the Guide for the Care and Use of Laboratory Animals, 8th edition
Animal Characteristics	SPS was optimized for group sizes of 6–8, age- and sex-matched	Handling frequency discussed in Methodological Considerations: Animal Handling Effects of age discussed in Methodological Considerations: Effects of Age Effects of sex discussed in Methodological Considerations: Effects of Sex Considerations for mice discussed in Methodological Considerations for SPS in Mice
	Handle animals prior to SPS to minimize effects of handling during experimental procedures; discussed in Methodological Considerations: Animal Handling	
Restraint (2 h)	Restrainers should be fitted correctly; see Methodological Considerations: Single Prolonged Stress: Restraint Stress	Restrainer type (hard plastic, decapicone, etc.); discussed in Methodological Considerations: Single Prolonged Stress: Restraint Stress
	Separate restrainers should be maintained for different age groups and/or sexes, and cleaned carefully between uses	
	Monitor animals to ensure they do not form a C-shape with their spine and become incapable of reorienting themselves as they may asphyxiate; see Methodological Considerations: Single Prolonged Stress: Restraint Stress	
	Restrain animals in a procedure room separate from housing rooms to avoid scent transfer	
Forced Swim (20 min)	Water temperature between 20 and 24°	Forced swim parameters (i.e., duration) will likely require optimization for females, younger animals, and different animal species/strains; see Methodological Considerations: Single Prolonged Stress: Forced Swim
	Container ~ 68 × 56 × 45 cm	
	Closely monitor animals during the forced swim; see Methodological Considerations: Single Prolonged Stress: Forced Swim	
	After the forced swim, dry animals as they are removed. Between groups, the swim container should be cleaned and refilled with fresh water	
Recovery Period (15 min)	Hyper- and hypo-thermia should be avoided; see Methodological Considerations: Single Prolonged Stress: Forced Swim	A heat source can be used; discussed in Methodological Considerations: Single Prolonged Stress: Forced Swim
Ether exposure (until loss of consciousness, ~5 min)	All procedures with ether, including animal exposure, should occur under a chemical fume hood for safety	Animals can be allowed to regain righting responses under a hood to enable ether evaporating from their fur to dissipate in the hood rather than in the home cage
	Ether should be placed below a vented floor, not in direct contact with animals, and ether vapors should be allowed to fill the ether chamber prior to the addition of animals	
	Closely monitor animals during ether exposure; see Methodological Considerations: Single Prolonged Stress: Ether	
	After induction of general anesthesia, promptly remove rats from the ether chamber	
7 day period following SPS-stressor exposure	Singly house animals for at least 7 days following SPS-stressor exposure; see Post SPS	Considerations for control animals in Control animals., and whether to house control and SPS animals in the same room and considerations for cage cleaning in Post SPS
	Animals should be “undisturbed” for 7 days following SPS-stressor exposure; discussed in Post SPS	

#### Methodological Considerations: Single Prolonged Stress: Restraint Stress

For the 2 h restraint stress, a key consideration is that the restrainer should be fitted correctly to prevent injury to the animal through struggling. If the selected restrainer is too large, the animal may turn its head toward its tail to form a C-shape with its spine. In this event, even if the tail is fixed in place, animals can struggle until they are incapable of reorienting and may asphyxiate. Restrainers that are too small can prevent respiration by inhibiting expansion of the chest. Thus, a correctly sized restrainer will prevent excessive movement and allow full expansion of the rib cage. Hard plastic restrainers are commercially available in a variety of sizes optimized for animals of different size ranges. If possible, separate restrainers should be maintained for different sexes and ages to account for size differences and potential scent transfer between sex and age groups. Additionally, animals should be restrained in a procedure room separate from housing rooms to avoid scent transfer. As a note, restrained animals often produce excessive fecal boli and urine, and placing disposable pads or paper towels beneath restrained animals will streamline cleaning of laboratory surfaces and restraints between uses. During restraint, rats can secrete red-colored porphyrin from glands surrounding their eyes and nose as a feature of their normal stress response, which should not be confused with blood (Mason et al., 2004). There are other restraint methods, including immobilization by fixing animals' limbs to a board, but the use of alternative restraint methods should be carefully considered and comprehensively reported. For example, compared with immobilization in a plastic restrainer, four limb prone restraint can prompt greater HPA-axis reactivity with differential rates of habituation in rats (Pitman et al., 1988).

#### Methodological Considerations: Single Prolonged Stress: Forced Swim

SPS was optimized for the forced swim to be conducted in groups of 6–8 adult male rats for 20 min, in a container ~68 × 56 × 45 cm, containing water at a temperature between 20 and 24°C. If females or other age groups are studied, forced swim groups should be age- and sex-matched to prevent excessive aggression. The forced swim must be closely monitored, as during the forced swim some animals may be temporarily held underwater by conspecifics. On rare occasions, an animal will sink under water due to exhaustion, distinguished from a controlled swim by the lack of limb movement and occasional exhalation of air bubbles without effort to return to the surface. In this event, the animal can be removed from the water for a 30 s rest before being gently returned to the water.

After the 20 min group forced swim, rats should be removed from the water promptly and dried as they are removed. Rats should then be provided with a 15 min recovery period. Access to a heat source during recovery, such as a surgical lamp or space heater (convection heater), will prevent hypothermia and can also be done in groups. To prevent overheating as a result of the heat source, excessively high temperatures (>26°C) should be avoided and rats should be provided with an option to escape the heat source. Signs that rats are overheating include bright pink coloration on their ears and hyperventilation (American College of Laboratory Animal Medicine Series, 2020).

Forced swim parameters will likely require optimization for females, younger animals, and different animal strains; the effects of forced swim are mediated by age-specific swimming gaits and somatic features including body fat percentage (buoyancy) and cardiovascular endurance (effort required), which can act as confounds across groups (Chen et al., 2015). Adult male rats, for which SPS was optimized, have a fat percentage as high as 27% (Tekus et al., 2018), whereas adult female rats can have ~8% body fat (with variability across age and strain; Dimitriou et al., 2000). Prior to puberty, male and female rats have equivalent average body fat of 12% (Engelbregt et al., 2001). For cross-species applications, mice swim at nearly half the speed of rats and exhibit ontogenetic differences in swimming gait and body composition, but compared with rats are generally less sexually dimorphic (with extensive variability across strains; Reed et al., 2007).

To reflect species differences in swimming propensity, when SPS procedures were applied to adult male mice and prairie voles, the forced swim was reduced by 10 and 15 min, respectively (Arai et al., 2016; Perrine et al., 2016). To offset the potentially reduced impact of the truncated forced swim, the mouse-SPS model includes 15 min of predator scent exposure (for more detail, see the Methodological Considerations for SPS in Mice section).

#### Methodological Considerations: Single Prolonged Stress: Ether

The final stressor in the SPS model, exposure to anhydrous diethyl ether, was optimized for group exposure of 6–8 adult male rats in a bell jar or desiccator with aqueous ether placed below a vented floor (internal diameter: 22 cm; ex. VWR cat. # 75871, vented floor purpose-built). Animals should be monitored closely during ether exposure, because overexposure can depress respiratory function or cause laryngospasm, ultimately leading to death (Brandstater and Eger, 1965). Induction of general anesthesia (loss of consciousness) can be verified by a lack of toe pinch or righting response. For personnel safety, ether exposure should be conducted in a chemical fume hood, and ether should be disposed of in accordance with institutional biosafety oversight. Additionally, a small volume of water can be added to the ether in the container to reduce volatility, which does not affect the ether's ability to induce loss of consciousness. After induction of general anesthesia, rats should be promptly removed from the ether chamber and placed in a recovery area in a fume hood. If animals are allowed to regain consciousness under a fume hood, the ether on their fur will evaporate into the hood, rather than in the caging room (potentially affecting animals outside the SPS treatment condition or personnel). Ether distinctly augments the impact of the SPS model compared with other anesthetics, because ether exposure triggers the release of adrenocorticotrophic hormone (ACTH), norepinephrine, epinephrine, and corticosterone in a time- and concentration-dependent manner in rats and mice (Cook et al., 1973; Glowa, 1993). Specifically, when other anesthetic agents have been used as a substitute for ether in the SPS protocol, effects on extinction retention were not observed (Knox et al., 2012b). Of note, ACTH and corticosterone responses to ether are present as early as 7 days of age in rats of both sexes, and are produced through activation of neural pathways distinct from those engaged by direct stress exposure (Matsuda et al., 1964; Raff et al., 2003).

#### Methodological Considerations for SPS in Mice

Preclinical PTSD research uses rats more than twice as often as mice, but transgenic mice are more available compared with transgenic rats such that mice are likely to become increasingly prevalent in preclinical psychiatric research (Török et al., 2019). Given that SPS was optimized for adult rats, which differ from mice in morphology of the brain, key features of stress response systems, and the effects of stress across domains including cognition, the application of SPS to mice and the translation of the results obtained in rats requires careful consideration (Armario and Castellanos, 1984; Schöner et al., 2017). There have been several studies that have modified SPS for application in mice (e.g., Wang et al., 2012; Yu et al., 2013; Tanaka et al., 2018; Teutsch et al., 2018); however, only one modified protocol has been demonstrated to replicate key outcome effects of the rat SPS model (Perrine et al., 2016). The mouse-SPS model described by Perrine et al. yields an extinction retention deficit and glucocorticoid receptor expression pattern similar to those detected following SPS in adult male rats. This mouse model of SPS has also been shown to blunt the behavioral sensitizing effects of ethanol, decrease striatal dopamine-2 receptor (D2) protein levels (Matchynski-Franks et al., 2016), increase immobility in a forced swim test (Malikowska et al., 2017), modify hippocampal serotonergic turnover in individuals with high fear generalization (Aikins et al., 2017), and reduce the percent of time spent in open arms of an elevated plus maze (Malikowska-Racia et al., 2020); it has also been used in the evaluation of pharmacological interventions (Malikowska-Racia et al., 2019; Azevedo et al., 2020).

In this model (Perrine et al., 2016), adult male mice are exposed to 2 h of restraint, which is unchanged from the rat protocol, but the choice of the restrainer type for mice is a BD Falcon© 50 ml conical tube with a screw-on top (with air holes located ~1/2 cm apart). The second stressor, the forced swim, is reduced by 10 min, reflecting the species-specific swimming capabilities of mice. Additionally, the water is room temperature (~23°C) and the volume of the group-forced swim tank is reduced to a 4 L plastic beaker. To increase the multimodality of the SPS paradigm, the mouse-SPS paradigm includes exposure to a predator scent (adult rat bedding) for 15 min. As with the rat SPS protocol, the final stressor is ether exposure until loss of consciousness. While rats are generally exposed to ether vapors through a ventilated floor in SPS, Perrine et al. used ether soaked cotton balls added at 1-min intervals to a standard microisolator polycarbonate cage without bedding in groups of ~8 adult males. The progressive addition enabled a slower onset of loss of consciousness more similar to the timing of the ether stressor in the rat paradigm. The quiescent period was unalerted from the 7 day period for the rat SPS model. Other approaches for modifying SPS for mice have included omitting the ether stressor (Yu et al., 2013), adding conditioned fear using foot shocks (Wang et al., 2012), and increasing the water temperature during the forced swim stressor (Tanaka et al., 2018; Teutsch et al., 2018).

***Pop-Out 3: Research personnel as part of an experimental context***.

*There is extensive evidence that laboratory rats discriminate between handlers, such that handlers of the same sex and approximate age can differentially affect learning measures (reviewed in Burn*, *2008**). Further, experimenters can serve as conditioned stimuli for learned associations, thereby becoming part of an experimental context and a necessary consideration for experimental design (e.g., Mumby et al.*, *1995**; Davis*, *2002**). An experimenter that becomes part of a fear-associated context, such as the context created during SPS or fear conditioning, may present a confound across SPS and non-SPS exposed groups or impair the extinction of learned fear. Therefore, the experimenter conducting the SPS procedures should ideally be a distinct individual from the experimenter conducting the subsequent behavioral testing, and the individual conducting fear conditioning procedures should be distinct from the person associated with the fear extinction context. If this is not possible, the person conducting the SPS procedures can administer the fear learning testing as a part of the fear-associated context but should not administer the extinction testing to avoid carry over effects of the SPS context. While this represents best practices, the effects of SPS on extinction retention have been detected in cases where there is personnel overlap between the SPS and fear learning procedures (Knox et al.*, *2016**; Noble et al.*, *2017**; Souza et al.*, *2017**)*.

As mice can show greater strain differences than rats, it should be noted that the mouse-SPS detailed in Perrine et al. was optimized for adult male C57Bl/6 mice and has also been applied to adult male Albino Swiss (CD-1) mice (Malikowska et al., 2017). As with rats, additional optimization of SPS procedures may be required for younger animals, females, and different strains. These modifications may reflect the body size, swimming capabilities, and stress responsivity of each group.

The variability in mouse-SPS protocols represented in the current literature further impedes meta-analyses and literature synthesis in traumatic stress models in mice. The use of standardized modifications to SPS methods for mice, such as the mouse-SPS model proposed in Perrine et al. could improve the robustness of SPS research in mice.

## Methodological Considerations for Fear Conditioning Following SPS

The effects of SPS have been tested on a multitude of outcome measures across a variety of biological systems. The diversity of research conducted using SPS and other preclinical models of PTSD speaks to the complex and diffuse outcomes of trauma that are of interest to preclinical researchers as a tool to inform clinical research. SPS effects on fear extinction retention have been evaluated in the context of HPA reactivity, glucocorticoid receptor expression and internalization, neuroinflammation, oxytocin and catecholamine levels, and sleep (Knox et al., 2012b; George et al., 2013; Keller et al., 2015b; Vanderheyden et al., 2015; Lin et al., 2016b; Wang et al., 2018; Chaby et al., 2019) (all represented in the current systematic analysis). Careful consideration of experimental conditions is essential because systematic differences in behavior of laboratory rodents across laboratories due to local conditions have been demonstrated to affect the quantification of freezing and anxiety-related behaviors (Crabbe et al., 1999; Meuth et al., 2013). Thus, consideration of differences in fear behaviors across species, sex, and age is essential for optimizing fear conditioning procedures and extinction retention testing. Here, we present considerations for optimizing extinction retention testing generated by the expert panel and systematic literature review. Fear conditioning is one of the most ubiquitous paradigms in behavioral neuroscience and is applied across a variety of experimental contexts (Beckers et al., 2013). The methodological considerations presented here to optimize fear conditioning as an SPS-outcome will not necessarily generalize to other applications of fear conditioning, therefore similar efforts to optimize fear conditioning in other contexts beyond SPS are encouraged (for example Wotjak, 2019).

The ability to retain fear extinction learning is of interest in part because it can facilitate recovery from trauma (Pitman et al., 2012). Further, there is clinical evidence to support that deficits in the retention of fear extinction are a feature of PTSD rather than a predisposing trait (Milad et al., 2008). Extinction retention deficits in PTSD may result from the inability to use safety cues to sustain suppression of extinguished fear memory (Garfinkel et al., 2014). Tests of extinction retention in humans use proxies of fear including skin conductance responses, fear potentiated startle, heart rate, pupil dilation, avoidance behavior, and verbal report (Lonsdorf et al., 2017, 2019). Contrastingly, in rodents, the current systematic review confirmed that measures of fear are generally restricted to freezing.

Limitations of extinction retention as an outcome measure arise from (i) constraints around interpreting freezing behavior as a proxy for fear such as confounding effects on locomotor activity, (ii) sex and age differences in the expression of fear behavior, and (iii) variation in operational definitions of extinction retention (Shansky, 2015; Bangasser and Wicks, 2017; Lonsdorf et al., 2019). For example, a fear behavior with higher prevalence in females, darting, is increasingly assessed and has methodological feasibility (Gruene et al., 2015). Indeed, automated behavioral assessment should not preclude examination of the behavior via videos to confirm that other defensive behaviors (e.g., escape behaviors such as running, jumping, vocalization, stereotyped head swaying) are not competing with freezing behavior and thus driving a potential underestimation of fear memory. Variability in the operational definition of extinction retention can also derive from methodological differences between preclinical and clinical extinction retention assays (Lonsdorf et al., 2019). For example, human paradigms often include the addition of a non-conditioned neutral stimulus (Greco and Liberzon, 2016; Risbrough et al., 2016; Lonsdorf et al., 2019). Additional details on the distinctions between fear conditioning procedures between humans and rats, as well as variability in definitions of extinction retention, are thoroughly described in Lonsdorf et al. (2017, 2019). Methodological considerations for the assessment of extinction retention in laboratory rats, optimized for male rats, are described below.

An extinction retention deficit is characterized by heightened fear expression during re-exposure to the conditioned stimulus in the extinction context, despite fear behavior having decreased over the course of extinction learning. In other words, when extinction retention is deficient, fear behavior is not suppressed upon re-exposure to the extinction context and returns to levels reminiscent of fear levels prior to extinction learning. Generally, fear conditioning, extinction training, and extinction retention testing are separated by 24 h and occur within the same time of day to account for circadian rhythms in stress responsivity systems and context learning processes (Cain et al., 2004; Atkinson et al., 2006). When extinction trials are administered immediately after fear conditioning, extinction learning can be suppressed in control animals; therefore it is important to separate fear conditioning procedures by 24 h in order to avoid extinction impairments that may mask effects of SPS (Maren, 2014). For cued extinction retention testing, it is critical to minimize fear of the context as measured during the baseline habituation period prior to the first cue presentation on the extinction retention testing day.

Behavior consistent with an extinction retention deficit could arise from several possible phenomena: failure to consolidate extinction learning, failure to retrieve extinction learning, or differential weighting of conflicting safety and fear associations that are both maintained and retrieved during extinction retention testing. The leading view is consistent with the latter phenomenon that the initial conditioned fear memory is not erased by extinction but is inhibited by a competing extinction memory, such that in a deficit of extinction retention, the fear memory is dominant (fear retention) but both learned associations are maintained.

### Considerations for Timing of Behavioral Testing

Of the SPS studies evaluated, 52% tested during the light phase but 48% did not specify when testing occurred (17/33 and 16/33, respectively). An increasing number of studies are conducting SPS procedures during the dark phase or using a reverse light/dark cycle to accommodate outcome testing during the active (dark) phase (Pooley et al., 2018a,b). Across preclinical research, there is increasing concern about the translational relevance of conducting behavioral assessments during the inactive (light) phase of the light/dark cycle (Castelhano-Carlos and Baumans, 2009; Verma et al., 2010). Such concerns are furthered by findings of (i) reduced cognitive performance in the light phase compared with the dark phase (Roedel et al., 2006), (ii) phase-by-sex interactions in anxiety-like and depression-like behavior and HPA axis reactivity (Verma et al., 2010), and (iii) phase-specific fear conditioning, extinction, and recall performance (Chaudhury and Colwell, 2002). Testing during the active phase can maximize translational relevance and avoid competing inactivity behaviors (Kopp, 2001). Given that there is variation on the testing phase in the current literature, there can be justifications to perform fear learning procedures in either the light or dark cycle following SPS. A key consideration for testing during the inactive phase is that fear learning tests generally rely on freezing behavior, which can be difficult to distinguish from inactivity, particularly for automated freezing detection software. This is especially challenging given findings that the inactivity rate is sex- and age-specific (reviewed in Rosenfeld, 2017). Increased inactivity may over-inflate freezing estimates, which may be exacerbated toward the end of a testing session as exploratory drives wane. Given the extensive circadian patterns in mammals, including activity and stress response systems that cause variations in responsiveness to the same stressors, light cycle details and time of day at testing should be reported and maintained within the same range for all animals in an experiment (Atkinson et al., 2006; Prager et al., 2011).

### Considerations for Conditioned Stimuli

When fear conditioning is an outcome measure following a stress procedure such as SPS, to avoid interactions between compounding stressors, conditioned stimuli should not be innately aversive (e.g., white noise elevates catecholamine and corticosterone levels in rats; De Boer et al., 1989). Additionally, conditioned stimuli should not overlap with stimuli present in the housing environment to prevent extinction outside of the experimental context (e.g., white noise, light). For example, white noise as a conditioned stimulus could interact with white noise produced by climate control systems in the housing environment. A pure auditory tone is recommended, and was used in 74% of cued fear conditioning studies following SPS (14 out of 19 studies in the systematic review). The expert panel concluded that variation in the tone used may have negligible effects on experimental outcomes, and efforts to optimize fear conditioning parameters are best focused on the amount of fear behavior exhibited by untreated, control animals, rather than directly replicating parameters from experiments published under different local conditions. Once fear conditioning parameters are optimized for local conditions, these parameters can be applied across all experiments conducted in these local conditions within a laboratory (but may need to be adjusted across groups that differ in animal features such as sex, age, strain, etc.). Variability in conditioned stimuli used following SPS are detailed in the systematic review; for example, tones varied from 10 to 30 s in duration; 1–9 kHz in frequency; and 70–80 dB in intensity. Of the 14 studies in the systematic review that utilized a tone, the majority used a 10 s tone (64%) at 2 kHz (57%) and 80 dB (79%).

### Considerations for Unconditioned Stimuli

The aversive stimulus used to evoke an unconditioned fear response was footshock in all studies evaluated here. Similarly, the use of footshock co-terminating with a pure auditory tone for fear conditioning following SPS was endorsed by the expert panel. As with conditioned stimuli variation, the consensus of the expert panel was that optimizing fear conditioning parameters is best achieved by focusing on behavior exhibited by control animals, rather than a direct replication of parameters from previous experiments that were conducted under distinct local conditions (see section Considerations for Optimizing Conditioned Fear Behavior). Local conditions shape behavior in fear learning tasks, including animal features (source, age, sex), time of day at testing, competing behaviors, test chamber size, baseline stress level, handling frequency, housing/husbandry details (temperature, lighting) (reviewed in Prager et al., 2011). Further, potential differences across testing equipment (chambers, recording devices) should also be considered when determining the required shock intensity and frequency and cue presentation number at the local level (Luyten et al., 2014). Reflecting these sources of variability, there was extensive variation in shock parameters used across the studies assessed in the systematic review (detailed in Table 1). For reference, conditioned stimuli used following SPS ranged from 1 to 10 in shock number; 0.3–1.5 mA in shock intensity; and 1–30 s in shock duration. The most prevalent shock features across the 33 studies in the systematic review were 5 shocks (33%) at 1 mA (52%) for 1 s (48%).

### Considerations for Optimizing Conditioned Fear Behavior

In order to discern group differences in fear learning, fear conditioning parameters must first be optimized in untreated, control animals. Optimizing fear conditioning parameters avoids ceiling effects (too much freezing) or floor effects (too little freezing) in the control group. Considerations to ensure fear conditioning parameters that provide robust data with the sensitivity to detect treatment effects are described in this section.

Prior to the first cue presentation, baseline freezing in the conditioning chamber should be assessed during each phase of fear learning. High levels of baseline freezing in untreated, control animals likely reflects sources of stress external to the experimental conditions, which may or may not be evident or noxious to humans (examples: inexperienced handlers, white noise, housing disturbances [vibrations, loud talking, doors slams], aversive smells in the housing room or on handlers, stress from transport to the behavioral room) or the continued presence of salient contextual cues that need to be removed. In the event of high baseline freezing, i.e., >30 percent time spent freezing prior to cue presentation, measures should be taken to minimize external sources of stress. Efforts to reduce baseline freezing can include: decreasing housing/husbandry disturbances, more frequent handling, retraining animal handlers, providing a longer acclimation period following transport to the behavioral testing room before placing animals in the fear conditioning chambers, etc. High baseline freezing specifically during the extinction retention phase could indicate that a feature of the extinction environment was aversive, which could arise, for example, from the use of white noise rather than a tone. If freezing is consistently high prior to cue presentation and throughout testing, it could reflect aversive features of the testing environment (vibrations, loud equipment, bright lights, etc.). Additionally, to ensure that animals can distinguish between contexts, contextual cues targeting a variety of sensory modalities should be used to differentiate the fear conditioning context from the extinction/extinction retention context. Examples of contextual cues include: the experimenter, chamber floor (texture or pattern insert in the second context), chamber wall pattern (color, pattern), odor (e.g., acetic acid vs. ammonium hydroxide), chamber doors (closed vs. open), and lighting (red light vs. white light). The design of the contextual cues should consider species-specific sensory capabilities. For example, laboratory rodents have limited visual acuity such that cues targeting olfactory or tactile modalities may be more salient (Artal et al., 1998).

**Figure 4:**
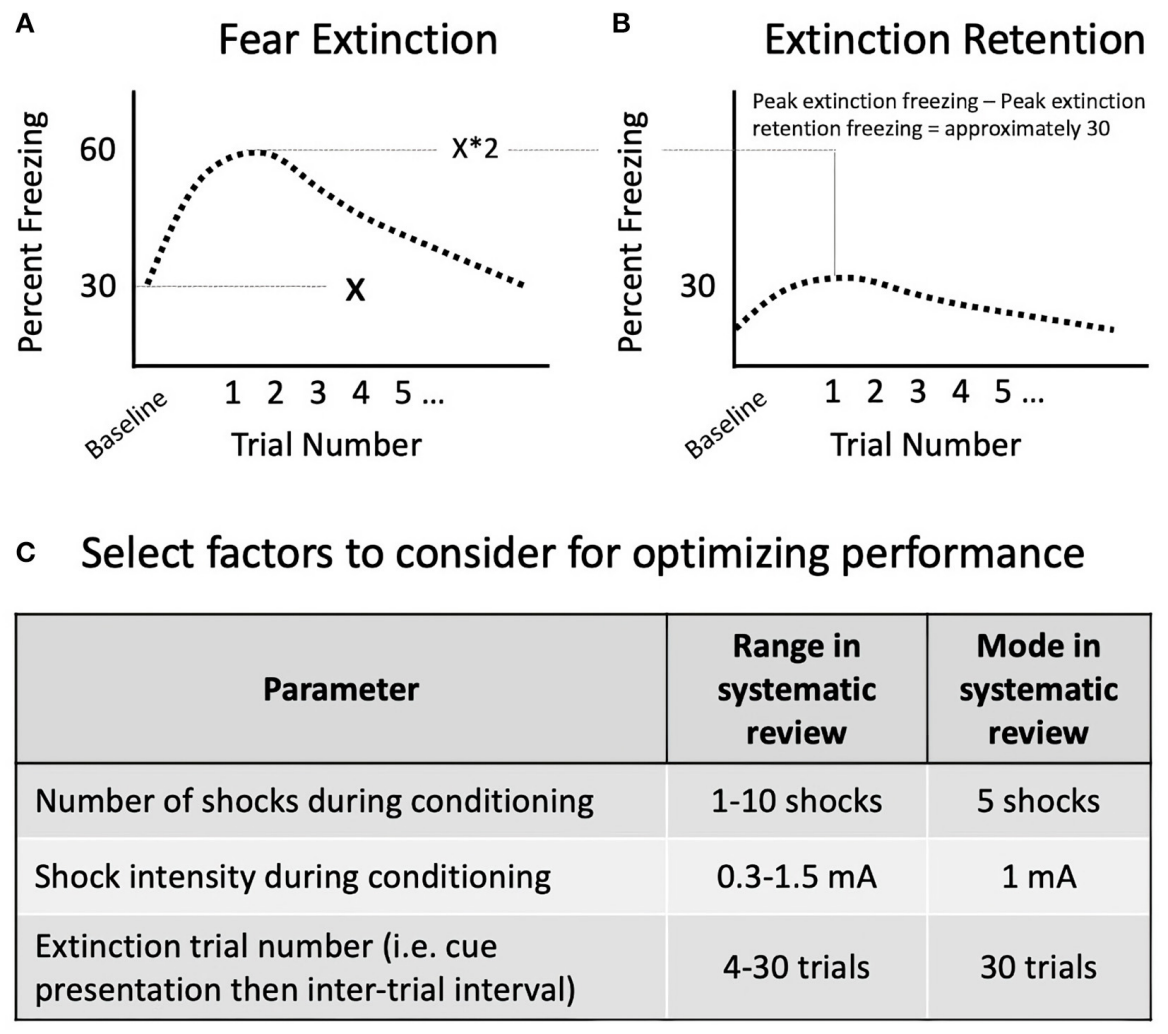
Hypothetical fear extinction **(A)** and extinction retention **(B)** performance that reflect appropriate local testing conditions and experimental parameters. Select parameters to consider for optimizing local conditions are presented in **(C)**, alongside variability and the most frequently used value from the publications in the systematic review. For a comprehensive discussion of optimizing local conditions see section Methodological Considerations for Fear Conditioning Following SPS. Under optimal experimental parameters, peak freezing during fear extinction training will be at least double the level of freezing detected at baseline before the first cue presentation. Freezing should not reach a ceiling effect (100% freezing) which would potentially mask a group difference. If consolidation of extinction learning is optimal, the difference between the peak freezing levels during extinction learning and extinction retention testing will be ~30, to ensure that extinction retention in untreated animals is sufficient to detect a deficit by comparison of groups.

During fear conditioning training, freezing generally increases over repeated cue-shock pairings and should end with high amounts of freezing without reaching a ceiling effect that could mask group differences. Conversely, too little freezing during fear conditioning could result in a floor effect during subsequent testing phases. Thus, freezing during fear conditioning should first be confirmed to be within previously published ranges for the sex, age, and strain studied. Following this, optimal consolidation of fear conditioning should be verified. For reference, select fear conditioning parameter ranges in the SPS studies discussed here are provided in Figure 4C. The consolidation of fear learning is reflected in the freezing response following the first 1–2 cue presentations during fear extinction; rats should show a peak freezing level early in fear extinction that indicates they retained the cue-shock association. As verification that fear conditioning was consolidated within a targeted range, the control group should show a mean peak freezing response (generally in the first 1–2 fear extinction trials) that is at least double the baseline percent freezing before the first cue presentation in fear extinction. Based on this relationship the minimum evidence of consolidation would be a baseline freezing level of 0–30% and peak freezing of 60–95% provided that the peak is at least double the baseline, is depicted in Figure 4A. If peak percent freezing is less than twice the baseline percent freezing, it could indicate that inadequate fear learning occurred, and the number or severity of shocks should be increased during the fear conditioning phase. Peak freezing is expected to vary based on local conditions (i.e., number of shocks, duration of FE), but peak freezing should not reach 100% (indicating a ceiling effect). If the peak freezing reaches a ceiling effect, it could indicate that the number or severity of shocks should be decreased to capture biological variation. Baseline freezing can range from 0 to 30%; excessive baseline freezing could suggest that the extinction context was not sufficiently altered from the fear learning context or that context generalization is occurring.

During the fear extinction phase, animals should decrease freezing behavior between the first and last cue presentation to ensure that extinction learning has taken place. If this condition is not met, extinction learning can be enhanced by increasing the number of cue presentations or extinction sessions (days) to lengthen the extinction training. Additional days of cue presentation can be useful in ensuring sufficient extinction, and is common outside of extinction retention tests (Matsumoto et al., 2008); however, for the current application, it may be challenging to contextualize an extinction retention deficit that is temporally inconsistent or transient and the neural mechanisms of extinction training in the amygdala and prefrontal cortex shift over time from inhibition to erasure of fear memory such that the duration of extinction training prior to extinction retention testing must be considered carefully (An et al., 2017). Optimal consolidation of fear extinction learning is necessary to ensure sensitivity to detect the effects of SPS. The strength of fear extinction consolidation is revealed in the first sessions of extinction retention. When fear extinction parameters are optimal, untreated control rats will decrease peak freezing during extinction retention by ~30% compared with peak freezing during extinction training (peak extinction—peak extinction retention = ~30% freezing); an example is depicted in Figure 4B. If this condition is met, then a treatment that completely prevented the retention of extinction would result in a 30% difference between groups during retention testing. If the difference between the mean peak freezing during fear extinction training and extinction retention in untreated rats is <30%, it could indicate that rats did not adequately extinguish the shock-cue pairing and fear extinction training should be lengthened. It should be noted that the optimal length of fear extinction training depends upon the experimental design; if reinstatement or fear renewal are endpoints rather than extinction retention, the optimal level of fear extinction would be more complete in order to examine these endpoints (Lonsdorf et al., 2019).

### Methodological Considerations for Optimizing Fear Learning in Mice

Fear behaviors are species specific; thus, optimization of fear conditioning procedures should reflect the specific fear behaviors exhibited by that species (Curzon et al., 2009). In addition to freezing, other behaviors that have been evaluated during fear conditioning procedures include darting, acoustic startle, operant suppression, avoidance, gaze-tracking, and heart rate (Chang et al., 2009). Additionally, fear associations in mice can be achieved with lower shock intensities and durations compared with rats (reviewed in Török et al., 2019). Parameters for fear conditioning following mouse-SPS are defined by Perrine et al. (2016), which include a reduced number of shock-tone pairings (3) and a reduced shock intensity (0.4 mA) compared with rats, which could serve as initial parameters for those looking to optimize their local conditions for fear conditioning following mouse-SPS. Additional considerations for fear conditioning procedures in mice are described in Wotjak (2019), including variations between mice strains in the optimal tone-shock intensity and number, variations in the pain threshold, and how to optimize the procedure for transgenic mice. In other models of severe stress, the delay before the emergence of PTSD-like symptoms is shorter in mice compared with rats, such that the timing of SPS outcome testing may need to be optimized for mice if expected effects are not detected (Török et al., 2019).

### Recommendations for Freezing Detection and Data Analysis

A challenge of fear conditioning paradigms can be quantification of freezing behavior. Currently, automated scoring systems have key limitations and handscoring requires expertise, time, introduces subjective judgement, and is incompatible with industrialization. Additionally, there is variation in operational definitions of freezing such that studies should report operational definitions of freezing behavior and all fear behaviors assessed. Whether freezing is scored using automated or manual methods, there are key methodological considerations, including considerations for video recording quality, that are described in this section and summarized in Table 3.

**Table 3:** Methodological considerations for detection and analysis of freezing behavior during fear learning procedures.

**Experimental Feature**	**Methodological Recommendations**	**Additional Considerations**
Video Recording	• Avoid adverse effects of video quality by recording trials at a minimum of 25 frames per second • Eliminate experimenter-induced variability (scent, movement, gender, etc.) by not having personnel in the behavioral testing room during trials • Mitigate data loss by using cloud-based backup for video storage	Additional features that can optimize video and data quality:• High contrast between the animal and the test background • Optimal lighting conditions (minimized shadows and glare, consistent lux value) • High image resolution • Maintain view of the entire animal in the frame (even while it is rearing) • Ensure the only in-frame movement is that of the animal
Manual Scoring of Freezing Behavior	• Use a detailed scoring protocol to standardize scoring across experiments and lab personnel turnover • Average scores from two independent, highly trained raters • Ensure raters are blinded to animal treatment condition • Continuous scoring may be more comprehensive than time sampling approaches	
Automated Scoring of Freezing Behavior	• Optimize threshold of detection for local lighting conditions then keep software settings consistent for all experimental animals • Background subtraction may enhance freezing accuracy	Automated scoring procedures and settings can be validated by correlating freezing data with corresponding data generated by manual scoring, see Recommendations for Freezing Detection and Data Analysis

For all efforts to quantify freezing, video recording quality can limit data quality. Handscoring and automated scoring quality can decline with low frame rates or low video quality (insufficient contrast between the rat and background, low color/gray scale depth, etc.; Haines and Chuang, 1993). The frames per second (fps) at which automated scoring is closest to handscoring is 30 fps (Anagnostaras et al., 2010). Additionally, some freezing detection software does not support videos <25 fps, which reflects the data sampling frequency, such that the longevity or generalizability of lower quality videos are more limited. Thus, it is recommended that video recordings be a minimum of 25 fps. Additional features that can optimize video quality, and thus data quality, are high contrast between the animal and the test background, optimal lighting conditions (minimized shadows and glare, consistent lux value), high image resolution, maintaining the entire animal in the frame (even while rearing), and ensuring the only in-frame movement is that of the animal.

To ensure the full scope of behavior is captured, videos should be analyzed continuously across each phase rather than with a time sampling approach. Data can then be blocked into time points within each testing phase. To ensure consistency with current SPS studies, each time point should include the cue presentation and the inter-trial interval for graphing and statistical analyses. Time points can be analyzed with a repeated measures analysis of variance within each testing phase. As fear conditioning experiments are limited by the number of testing chambers, testing across several waves/cohorts is generally necessary. Cohort effects can be strong, and care should be taken to compare cohorts before combining cohort data to determine whether external factors may have influenced the integrity of a cohort.

The use of automated software can standardize scoring methods and increase throughput, but researchers must ensure that software freezing data are highly correlated with hand-scored freezing data generated by an experienced experimenter. To do this, a subset of videos should be hand-scored and the data should be correlated with data from the selected software method. Procedures for titrating automated scoring parameters to best reflect manual scoring are provided in Anagnostaras et al. (2010). Thresholds for freezing detection should be optimized to match handscoring under each experimental context (lighting, background, camera resolution, etc.), such that different thresholds may be needed for fear conditioning and extinction/extinction retention contexts (Pham et al., 2009). Experimenters should report the software version used, as well as internal thresholds for freezing detection and duration, given that thresholds can determine whether group differences are detected statistically (Luyten et al., 2014). Background subtraction or dynamic background subtraction can increase the accuracy of freezing detection software and is recommended to prevent the testing background (including urine and fecal boli produced during the test) from interfering with detection of the animal's movement (Marchand et al., 2003; Anagnostaras et al., 2010). Without background subtraction, video-scoring software underestimates freezing near 0% time spent freezing and overestimates freezing near 100% freezing (Marchand et al., 2003). Automated systems that use photobeams, with detectors placed 13 mm or more apart, may not have the spatial resolution necessary to detect small movements (such as minor grooming or head swaying) and may assess immobility rather than freezing (Marchand et al., 2003; Anagnostaras et al., 2010). There are other methods in use to measure freezing, including recording a rat's motor activity through displacement of their testing chamber on a specialized load-cell platform (Marek et al., 2018), but a comprehensive assessment of freezing measures is beyond the scope of this work.

If handscoring is used, to minimize subjectivity, videos should be scored by two researchers blind to treatment condition and their scores should be averaged. Additionally, a detailed scoring protocol should be used to standardize scoring across experiments and lab personnel turnover. Handscoring may provide advantages for integrating additional fear behaviors that may be essential for the application of fear learning to females or younger animals (Graham et al., 2018). Commercially available automated methods have yet to be optimized for fear behaviors beyond freezing, but key progress is underway in defining and automating the detection of darting as a fear behavior in females.

### Recommendations for Freezing Behavioral Analysis: Subject Selection Using Learning Criteria

While the majority of SPS studies do not remove animals based on learning performance, 15% of the SPS studies evaluated here did report using cut off scores to remove animals designated as poor learners. The reasoning for this approach is that extinction retention cannot be measured in animals that do not first show adequate fear conditioning and fear extinction. Using learning criteria, however, can be problematic as criteria are highly variable across laboratories; thus, if they are used, transparency is critical to support the replicability of findings. Additionally, unexpectedly low levels of freezing could indicate that animals are exhibiting different fear behaviors, and researchers should consider evaluating additional behaviors (e.g., escape directed behavior). Exclusion criteria have been used to eliminate animals with abnormal baseline fear behavior (e.g., time spent freezing before the conditioned stimulus >50%; Broadwater and Spear, 2014 or 100%; Storsve et al., 2010), low fear learning performance (e.g., freezing below 30% during the last block of fear conditioning; Storsve et al., 2010 or the first block of extinction; McCallum et al., 2010), low extinction learning performance (e.g., freezing that does not decrease between the initial and final extinction block; Abraham et al., 2012), and low extinction retention performance (based on variation from a group mean (Knox et al., 2016). If learning criteria are applied, they should not disproportionately exclude rats from a specific treatment group. Any rats excluded, and the treatment groups to which they were assigned, and ideally a sensitivity analysis (data with and without excluded data) should be included. If more than a few rats must be excluded because they failed to meet learning criteria, it could indicate that fear learning parameters are not optimized and should be evaluated (Section Methodological Considerations: Effects of Age).

## Conclusions

There is currently ample evidence that the reproducibility of research findings is poor across nearly all scientific disciplines (reviewed in Ioannidis, 2005). While the sources of poor reproducibility, or the ability to produce similar scientific results through independent replication using the same methodology within the same laboratory or across laboratories, are numerous and controversial, they include such phenomena as a lack of scientific rigor, low statistical power, positive publication bias, a lack of preplanned statistical analyses, and poor or incomplete reporting of methodological detail in accordance with the ARRIVE 2.0 guidelines, among others (Goodman et al., 2016). Pushes for increased standardization of the applied experimental method, such as that detailed in this study, will not remove all sources of phenotypic variation within a given model as natural biological variation does exist and is an important part of accessing a given model's potential for extrapolating the findings to another species, primarily humans (Voelkl et al., 2020). However, meaningful conclusions about the robustness of a given outcome to an experimental manipulation, i.e., the application of the SPS model and its subsequent effect on conditioned fear, cannot be reliably determined when the model itself is applied under such wide ranging methodological variation or when publications fail to report all of the methodological detail required to independently replicate the findings and draw cross-study conclusions. Replication based on methodological rigor requires the identification of those conditions that need to be sufficiently mimicked to assess prior claims and thus build knowledge. This is particularly relevant for models of stress-induced phenotypes, such as those attempting to model PTSD, as the actual neural bases of these disorders is currently unknown and presents considerable heterogeneity in terms of the complexity of symptom presentation and severity, trauma type, comorbidities, and demographic features (reviewed in Galatzer-Levy and Bryant, 2013). Thus, efforts toward rigor, detailed methodological reporting, and increased methodological consensus are essential across all preclinical models, and particularly in PTSD animal models, to provide a foundation for studying the biological underpinning of these disorders and allow for the application of statistical approaches that facilitate the drawing of conclusions across studies (i.e., meta-analyses). In the SPS studies evaluated here (*n* = 33), methodological reporting was incomplete across all domains evaluated, and attempts to clarify key methodological details by contacting the lead or corresponding authors was only possible in 42% (14/33) of the included studies. This is important, as studies are often excluded from meta-analyses and systematic reviews due to incomplete reporting of key study details, which limits the generalization of these analyses and can lead to false avenues of investigation in future studies. This emphasizes the need for more comprehensive methodological reporting and open communication among researchers. Frequently omitted details spanned animal features across the 33 studies evaluated: (single vs. group housing, 30%), SPS methods (single vs. group application of forced swim, 67%; restrainer type, 52%), and fear conditioning methods (light/dark phase at testing, 48%; manual vs. automated scoring, 15%; continuous vs. time sampling, 33%).

There are some limitations to this study. First, the recommendations presented in this paper are limited to SPS and the use of fear conditioning with SPS and may not generalize to other applications of fear conditioning. However, there is no reason to suggest that the challenges outlined here are confined to the SPS model, and similar efforts to facilitate reproducibility through detailed methodological discussions should be conducted for other preclinical models of stress-induced pathology. Second, this paper does not address the relationship between the optimized SPS parameters and the potential extinction retention deficits. To our knowledge, there are not yet data that address this relationship and therefore, it is not yet possible to integrate empirically-based understanding of this relationship into this formal context; this data should be collected in the future. Further, while standardized, comprehensive methodological reporting across all preclinical experiments can the enhance replicability and robustness of pre-clinically-derived results under a specific context and thereby the utility of preclinical research to build critical knowledge on basic biological mechanisms, the use of preclinical models for translational research will be limited until preclinical models are validated under conditions that account for the natural phenotypic and environmental variations observed in humans. Thus, care must be taken to optimize the use of preclinical models to the extent that is possible and then apply those optimized parameters across, for example, different strains, sexes, or environmental parameters, to determine whether the model holds true translational potential for the human condition. We suggest that the current recommendations derived from variation in published SPS designs can facilitate initial efforts aimed at improving reproducibility and pinpoint the features which are most crucial in order to maximize robustness of SPS studies, which can then form the basis for future translational efforts (Rueda et al., 2020). Finally, recent developments in the genetics of PTSD offer new opportunities to model disease based on direct readouts from human data and identified pathogenic pathways (Bespalov and Steckler, 2018).

## Data Availability Statement

The data supporting the conclusions of this article will be made available by the authors upon reasonable request.

## Author Contributions

CF-B designed the search strategy, conducted the systematic review, organized/summarized the workshop recommendations, and wrote and edited the manuscript. LC helped conduct the systematic review, performed the data analysis, and wrote and edited the manuscript. ND, DK, IL, ML, CM, SP, VR, and ES contributed to the workshop recommendations and edited the manuscript. AJ and MH provided project oversight and contributed to the writing and editing of the manuscript. All authors have read and approved the final version of the manuscript.

## Conflict of Interest

CF-B, LC, AJ, and MH are employed by Cohen Veterans Bioscience, a nonprofit 501(c)(3) research organization. ND has held a part-time paid position at Cohen Veterans Bioscience, has been a consultant for Sunovion Pharmaceuticals and is on the scientific advisory board for Sentio Solutions. The remaining authors declare that the research was conducted in the absence of any commercial or financial relationships that could be construed as a potential conflict of interest.

## Abbreviations

SPS: Single Prolonged Stress
CNS: Central Nervous System
PTSD: Posttraumatic Stress Disorder
HPA: Hypothalamic-Pituitary-Adrenal.
