# Supplementary material for: Systematic Review and Methodological Considerations for the Use of Single Prolonged Stress and Fear Extinction Retention in Rodents
Source: Front Behav Neurosci. 2021 May 14;15:652636. doi: 10.3389/fnbeh.2021.652636 (PMC8162789; doi:10.3389/fnbeh.2021.652636)
Supplement: Supplementary file 1 [file Table_1.DOCX]

**Supplementary Material**

**Supplementary Table 1**: Studies Included in SPS Systematic Review

| Author | Year | Title |
| --- | --- | --- |
| Imanaka et al. | 2006 | Importance of early environment in the development of post-traumatic stress disorder-like behaviors |
| Takahashi et al. | 2006 | Effect of paroxetine on enhanced contextual fear induced by single prolonged stress in rats |
| Khoda et al. | 2007 | Glucocorticoid receptor activation is involved in producing abnormal phenotypes of single-prolonged stress rats: a putative post-traumatic stress disorder model |
| Iwamoto et al. | 2007 | Single prolonged stress increases contextual freezing and the expression of glycine transporter 1 and vesicle-associated membrane protein 2 mRNA in the hippocampus of rats |
| Harada et al. | 2008 | Activation of the serotonin 5-HT2C receptor is involved in the enhanced anxiety in rats after single-prolonged stress |
| Yamamoto et al. | 2008 | Effects of single prolonged stress and D-cycloserine on contextual fear extinction and hippocampal NMDA receptor expression in a rat model of PTSD |
| Yamamoto et al. | 2010 | Alterations in the hippocampal glycinergic system in an animal model of posttraumatic stress disorder |
| Takei et al. | 2011 | Enhanced hippocampal BDNF/TrkB signaling in response to fear conditioning in an animal model of posttraumatic stress disorder |
| Knox et al. | 2012 | Single prolonged stress disrupts retention of extinguished fear in rats |
| Knox et al. | 2012 | Glucocorticoid receptors and extinction retention deficits in the single prolonged stress model |
| Matsumoto et al. | 2013 | Vorinostat ameliorates impaired fear extinction possibly via the hippocampal NMDA-CaMKII pathway in an animal model of posttraumatic stress disorder |
| Eskandarian et al. | 2013 | Effects of systemic administration of oxytocin on contextual fear extinction in a rat model of post-traumatic stress disorder |
| Mirshekar et al. | 2013 | Systemic administrations of beta-estradiol alleviate both conditioned and sensitized fear responses in an ovariectomized rat model of post-traumatic stress disorder |
| Xia et al. | 2013 | FGF2 blocks PTSD symptoms via an astrocyte-based mechanism |
| Miao et al. | 2014 | Midazolam ameliorates the behavior deficits of a rat posttraumatic stress disorder model through dual 18 kDa translocator protein and central benzodiazepine receptor and neurosteroidogenesis |
| George et al. | 2015 | The effect of chronic phenytoin administration on single prolonged stress induced extinction retention deficits and glucocorticoid upregulation in the rat medial prefrontal |
| Keller et al. | 2015 | Inhibiting corticosterone synthesis during fear memory formation exacerbates cued fear extinction memory deficits within the single prolonged stress model |
| Feng et al. | 2015 | FGF2 alleviates PTSD symptoms in rats by restoring GLAST function in astrocytes via the JAK/STAT pathway |
| Vandereyden et al. | 2015 | Sleep alterations following exposure to stress predict fear-associated memory impairments in a rodent model of PTSD |
| Keller et al. | 2015 | Sex differences in the single prolonged stress model |
| Liu et al. | 2016 | NOX2 Mediated-Parvalbumin Interneuron Loss Might Contribute to Anxiety-Like and Enhanced Fear Learning Behavior in a Rat Model of Post-Traumatic Stress Disorder |
| Lin et al. | 2016 | Traumatic stress causes distinctive effects on fear circuit catecholamines and the fear extinction profile in a rodent model of posttraumatic stress disorder |
| Knox et al. | 2016 | Neural circuits via which single prolonged stress exposure leads to fear extinction retention deficits |
| Lin et al. | 2016 | Escitalopram reversed the traumatic stress-induced depressed and anxiety-like symptoms but not the deficits of fear memory |
| Noble et al. | 2017 | Effects of vagus nerve stimulation on extinction of conditioned fear and post-traumatic stress disorder symptoms in rats |
| Han et al. | 2017 | Change of Rin1 and stathmin in the animal model of traumatic stresses |
| Wang et al. | 2018 | Effects of oxytocin on fear memory and neuroinflammation in a rodent model of posttraumatic stress disorder |
| Moulton et al. | 2018 | Characterizing changes in glucocorticoid receptor internalization in the fear circuit in an animal model of post traumatic stress disorder |
| Chen et al. | 2018 | Effects of Trauma in Adulthood and Adolescence on Fear Extinction and Extinction Retention: Advancing Animal Models of Posttraumatic Stress Disorder |
| Chaby et al. | 2019 | Cognitive Flexibility Training Improves Extinction Retention Memory and Enhances Cortical Dopamine With and Without Traumatic Stress Exposure |
| Lin et al. | 2019 | Hyperbaric oxygen therapy restored traumatic stress-induced dysregulation of fear memory and related neurochemical abnormalities |
| Lin et al. | 2019 | Subchronic administration of aripiprazole improves fear extinction retrieval of Pavlovian conditioning paradigm in rats experiencing psychological trauma |
| RaiseAbdullahi et al. | 2019 | Time-dependent protective effects of morphine against behavioral and morphological deficits in an animal model of posttraumatic stress disorder. |
